# Functional cooperation between co-amplified genes promotes aggressive phenotypes of HER2-positive breast cancer

**DOI:** 10.1016/j.celrep.2021.108822

**Published:** 2021-03-09

**Authors:** Yongguang Yang, Marissa Leonard, Zhenhua Luo, Syn Yeo, Gregory Bick, Mingang Hao, Chunmiao Cai, Mahmoud Charif, Jiang Wang, Jun-Lin Guan, Elyse E. Lower, Xiaoting Zhang

**Affiliations:** 1 Department of Cancer Biology, University of Cincinnati College of Medicine, Cincinnati, OH 45267, USA; 2 Graduate Program in Cancer and Cell Biology, Vontz Center for Molecular Studies, University of Cincinnati College of Medicine, Cincinnati, OH 45267, USA; 3 The Liver Care Center and Divisions of Gastroenterology, Hepatology, and Nutrition, Cincinnati Children’s Hospital Medical Center, Cincinnati, OH 45229, USA; 4 Department of Internal Medicine, University of Cincinnati College of Medicine, Cincinnati, OH 45267, USA; 5 Department of Pathology and Laboratory Medicine, University of Cincinnati College of Medicine, Cincinnati, OH 45267, USA; 6 University of Cincinnati Cancer Center, University of Cincinnati College of Medicine, Cincinnati, OH 45267, USA; 7 Lead contact

## Abstract

MED1 (mediator subunit 1)co-amplifies with HER2, but its role in HER2-driven mammary tumorigenesis is still unknown. Here, we generate MED1 mammary-specific overexpression mice and cross them with mouse mammary tumor virus (MMTV)-HER2 mice. We observe significantly promoted onset, growth, metastasis, and multiplicity of HER2 tumors by MED1 overexpression. Further studies reveal critical roles for MED1 in epithelial-mesenchymal transition, cancer stem cell formation, and response to anti-HER2 therapy. Mechanistically, RNA sequencing (RNA-seq) transcriptome analyses and clinical sample correlation studies identify Jab1, a component of the COP9 signalosome complex, as the key direct target gene of MED1 contributing to these processes. Further studies reveal that Jab1 can also reciprocally regulate the stability and transcriptional activity of MED1. Together, our findings support a functional cooperation between these co-amplified genes in HER2^+^ mammary tumorigenesis and their potential usage as therapeutic targets for the treatment of HER2^+^ breast cancers.

## In brief

In this study, Yang et al. generate a more clinically relevant MMTV-HER2/MMTV-MED1 mammary tumor mouse model and discover the critical roles and molecular mechanisms of MED1 overexpression in mediating the aggressive phenotypes of HER2^+^ tumor progression, metastasis, cancer stem cell formation, and therapy resistance.

## Graphical Abstract

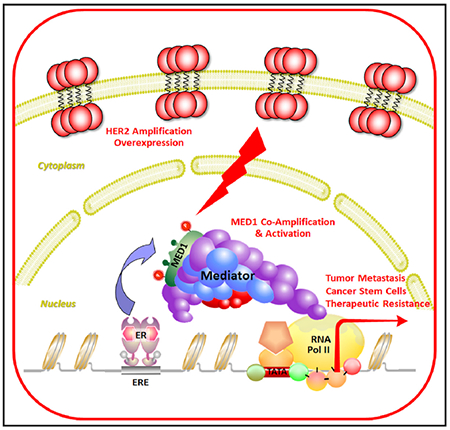

## INTRODUCTION

Breast cancer can be largely divided into multiple subtypes (e.g., estrogen receptor-positive [ER^+^] luminal A, ER^+^ luminal B, human epidermal growth factor receptor 2-positive [HER2^+^] and triple negative) based on gene expression profile analyses (Perou et al., 2000; Cancer Genome Atlas Network, 2012). The HER2/neu receptor is an epidermal growth factor (EGF) family transmembrane tyrosine kinase that is amplified and overexpressed in 20%–30% of breast cancers (Yarden and Sliwkowski, 2001; Zhou and Hung, 2003). Despite recent advances in developing effective anti-HER2 therapies, many patients still relapse after treatment because of resistance (Martin and López-Tarruella, 2016; Fabi et al., 2016). Clinically, at least 50% of HER2-overexpressing breast cancers are ER^+^, and this breast cancer subtype is particularly challenging to treat due to its resistance to both anti-estrogen and anti-HER2 therapies (Arpino et al., 2004; Nahta et al., 2006; Chen et al., 2008; Rexer and Arteaga, 2012). A better understanding of the HER2/ER axis and the key downstream pathways involved in HER2-driven breast tumorigenesis is important because it could potentially provide strategies for future breast cancer treatment.

ER is the key functional mediator of estrogen and plays prominent roles in breast cancer (Gruber et al., 2002; Yager and Davidson, 2006). Estrogen antagonists can effectively slow the growth of breast tumors and are used for prevention and treatment in ER^+^ breast cancer patients (Park and Jordan, 2002; O’Shaughnessy, 2006). However, HER2 amplification has been associated with aggressive disease, poor prognosis, and resistance to anti-estrogen therapy of ER^+^ breast cancer (Slamon et al., 1987; Osborne and Schiff, 2011). There is increasing evidence for the existence of multiple levels of crosstalk between ERα and HER2 signaling pathways in breast cancers, which lead to tumor progression and therapy resistance (Osborne and Schiff, 2011; Montemurro et al., 2013).

The mediator complex has proven to be the main hub for direct communication between transcriptional activators and the general transcription machinery (Malik and Roeder, 2000; Roeder, 2003). Recent studies have established MED1 (Mediator Subunit 1, also named TRAP220, DRIP205, or MED220) as a key transcriptional coactivator for ERα during both normal mammary gland development and breast tumorigenesis (Zhang et al., 2005a; Jiang et al., 2010; Lonard and O’malley, 2007). Significantly, the *MED1* gene is located at the chromosome *17q12* region, also known as the HER2 amplicon, and co-amplifies with HER2 in almost all instances in breast cancer (Luoh, 2002; Sahlberg et al., 2013). We have recently further confirmed MED1 overexpression and its correlation with HER2 status at the protein level using human breast cancer tissue microarrays (Cui et al., 2012). Importantly, we found that MED1 serves as a key crosstalk point for the HER2 and ER α pathways in regulating both ERα-mediated transcription and resistance of breast cancer cells to anti-estrogen therapies (Cui et al., 2012).

Our study further indicated a causal relationship between HER2 overexpression and MED1 phosphorylation, promoter recruitment, and activation of ER target genes. Importantly, MED1 knockdown significantly sensitized otherwise resistant HER2-overexpressing human breast cancer cells to anti-estrogen treatments, such as tamoxifen and fulvestrant (Cui et al., 2012; Zhang et al., 2013). Aside from clinical data showing the correlation of high MED1 levels with poor outcome and disease-free survival of patients undergoing anti-estrogen therapies (Ross-Innes et al., 2012; Nagalingam et al., 2012), a recently published prominent study also discovered an increased frequency of MED1 mutation in circulating tumor cells (CTCs) in breast cancer patients following anti-estrogen and anti-HER2 treatments (Murtaza et al., 2013). However, despite these discoveries, whether the overexpression of MED1 may play a role in HER2-driven tumorigenesis or treatment resistance is still unknown.

To address that, we have generated MED1 mammary-specific overexpression mice and crossed them to a mouse mammary tumor virus (MMTV)-HER2 mammary tumor mouse model in this study (Guy et al., 1992). Although MED1 overexpression itself does not induce mammary tumor formation, we observed significantly promoted tumor onset, growth, metastasis, and elevated primary tumor multiplicity in MMTV-HER2/MMTV-MED1 double-transgenic mice. Our studies further revealed that MED1 overexpression promotes epithelial-to-mesenchymal transition (EMT), cancer stem cell (CSC) formation, and the resistance to anti-HER2 therapy of MMTV-HER2 tumors. Moreover, our RNA sequencing (RNA-seq) transcriptome analyses and clinical specimen correlation studies have revealed signalosome complex protein Jab1 as a direct MED1 overexpression regulated target gene that plays critical roles in the above phenotype observed. Finally, we found that Jab1 can also reciprocally regulate the stability and transcriptional activity of MED1 through controlling its ubiquitin-proteasome pathway-mediated turnover and cyclic recruitment at target gene promoters. Together, our data revealed key roles for the MED1/Jab1 axis in HER2-driven mammary tumorigenesis and its potential usage as a therapeutic target for the treatment of HER2 and ER double-positive luminal B subtypes of breast cancer.

## RESULTS

### Generation and characterization of mammary-specific MED1 overexpression mice

**Figure 1.**
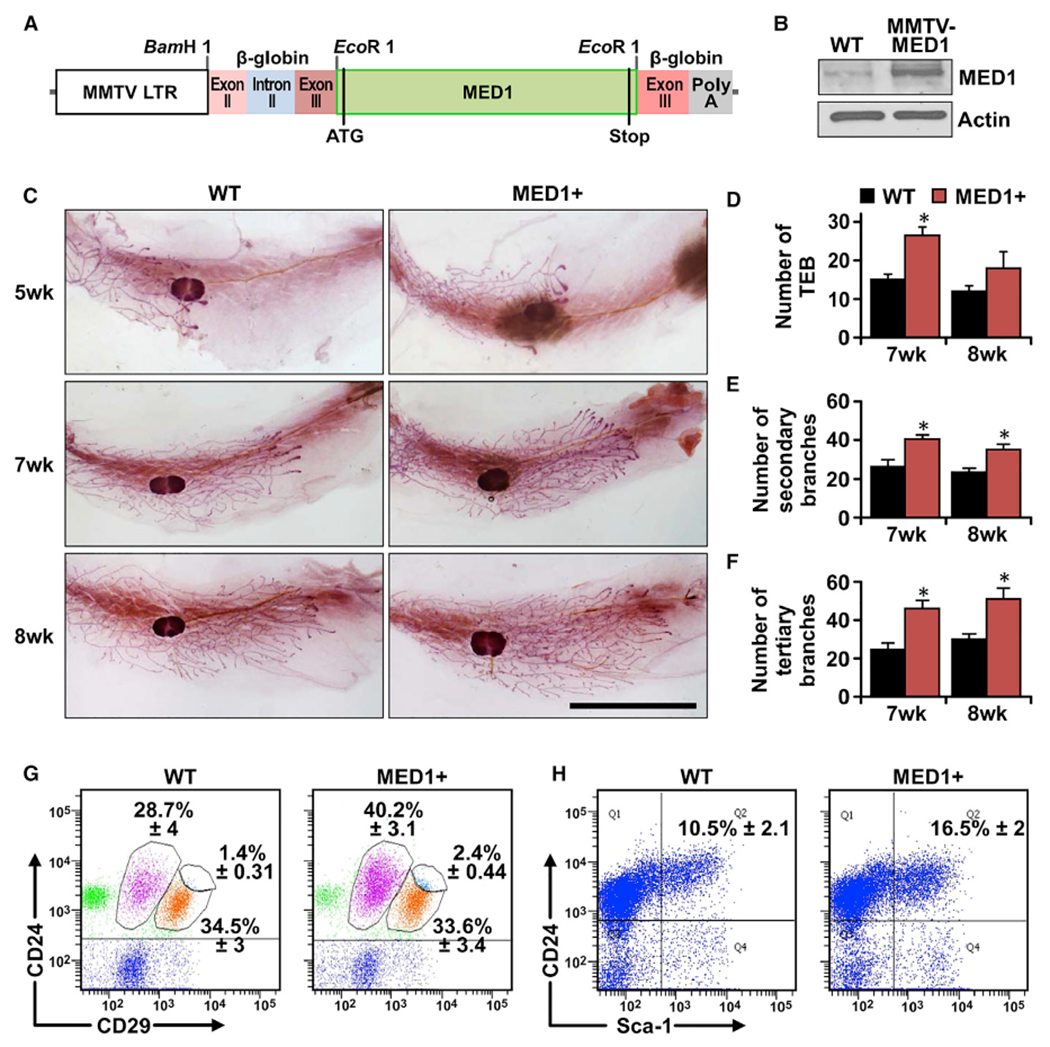
Generation and characterization of mammary-gland-specific MED1-overexpression mice (A) Schematic representation of MED1-overexpression transgenic construct (MMTV-MED1). (B) MED1 expression in mammary epithelial cells of MMTV-MED1 transgenic mouse was assessed by immunoblotting. (C) Whole-mount staining of pubertal mammary gland at age of 5, 7, and 8 weeks. Scale bar: 1 cm. (D–F) Analyses of the number of terminal end buds (TEBs) (D), secondary branches (E), and tertiary branches (F) of mice in (C). (G) Mammary cells from 7-week-old wild-type (WT) and MMTV-MED1 mice were analyzed by flow cytometry using antibodies against cell surface markers Lin (CD31, CD45, and Ter119), CD24, and CD29. (H) Flow cytometry analyses of Lin^−^CD24^+^Sca1^+^ cells in mammary glands of 7-week-old WT and MMTV-MED1 mice. The values are obtained from three independent experiments and shown as mean ± SD. *p < 0.05.

To study the role of MED1 overexpression in HER2-mediated tumorigenesis *in vivo*, we first generated an *MMTV-MED1* transgenic mouse model overexpressing MED1 under the control of *MMTV* long-terminal repeat promoter/enhancer (Pierce et al., 1993) (Figure 1A). We obtained a total of six transgenic lines, with lines 2 and 6 expressing higher levels of MED1 protein in the mammary gland and exhibiting a very similar phenotype. As shown in Figures 1B–1F for the line 2 mice, whole-mount staining indicated that MED1 overexpression only slightly accelerated pubertal mammary gland development in terms of the number of terminal end buds (TEBs) and secondary and tertiary branches. To determine whether MED1 overexpression affects mammary stem/progenitor cell formation, we carried out flow cytometry analysis using antibodies against established markers CD24, CD29, and Lin (CD31, CD45, and Ter119) (Figure 1G) (Shackleton et al., 2006; Visvader, 2009). We found that the Lin^−^CD24^+^CD29^lo^ luminal cell and Lin^−^CD24^+^CD29^hi^ mammary gland stem cell (MaSC)-enriched populations were increased in transgenic mammary glands (Figures S1A and S1B), but the Lin^−^CD24^+^CD29^med^ basal epithelial cells were not affected by MED1 overexpression (Figure S1C). Furthermore, we detected an increased Lin^−^CD24^+^Sca1^+^ cell population in transgenic mammary gland (Figures 1H and S1D), suggesting an increase in ER^+^ luminal progenitor cells (Sleeman et al., 2007; Welm et al., 2002). Consistent with that, immunohistochemical (IHC) staining detected an increased percentage of Ki67-positive proliferating cells and ERα-positive epithelial cells in MMTV-MED1 transgenic mammary gland compared with that of wild-type controls (Figures S1E–S1G). Other than these, we found the MMTV-MED1 transgenic mice are overtly normal with respect to fertility, pregnancy, and lactation, with no mammary hyperplasia or tumor formation observed up to 2 years of age.

### Overexpression of MED1 promotes MMTV-HER2 mammary tumorigenesis

**Figure 2.**
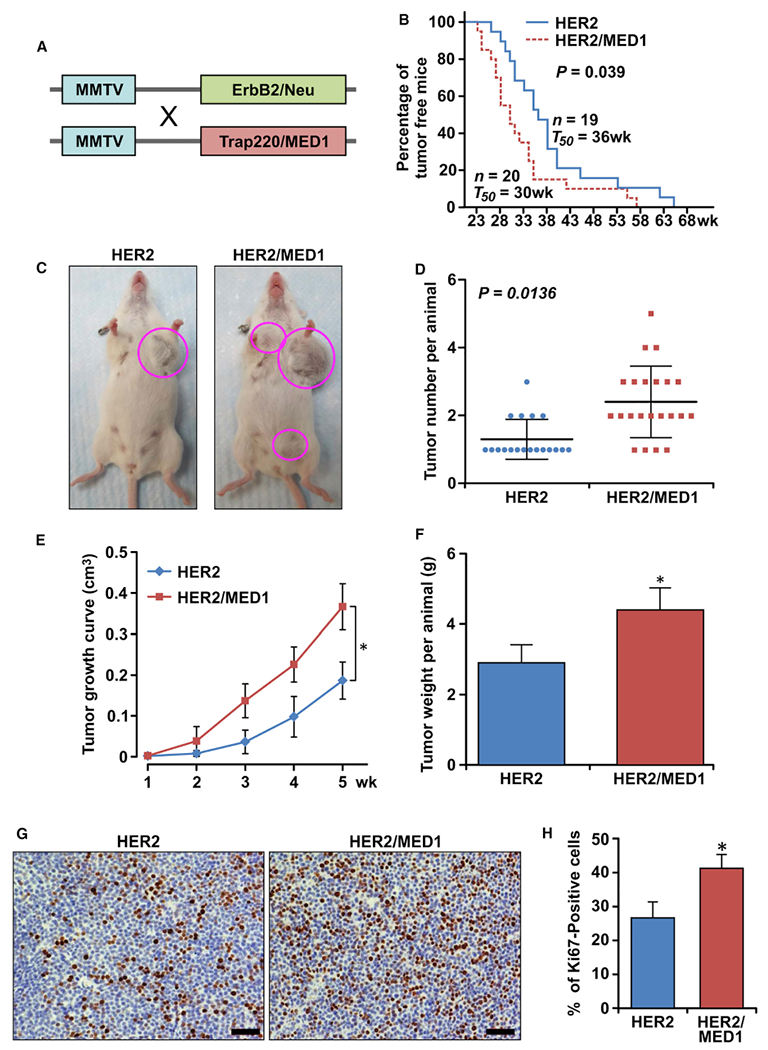
Overexpression of MED1 greatly enhances MMTV-HER2 mammary tumorigenesis (A) Schematic representation of generation of MMTV-HER2/MMTV-MED1 double-transgenic mice by crossing MMTV-MED1 and MMTV-HER2 mouse models. (B) Kaplan-Meier analysis of tumor onsets with the median tumor onset value T_50_. (C) Representative image of MMTV-HER2 and MMTV-HER2/MMTV-MED1 tumor-bearing mice at 40 weeks. (D) Quantification of total tumor numbers per mouse in each group. (E and F) Tumor growth curves (E) and total tumor weight per mouse (F) in MMTV-HER2 and MMTV-HER2/MMTV-MED1 groups. (G and H) IHC analyses of Ki67 expression in MMTV-HER2 and MMTV-HER2/MMTV-MED1 tumor sections (G) and quantifications (H). Scale bar: 50 μm. The values are obtained from three independent experiments and shown as mean ± SD. *p < 0.05.

To further examine whether overexpression of MED1 plays a role in HER2-mediated mammary tumorigenesis, we crossed MMTV-MED1 mice with MMTV-HER2 mice (Figure 2A). Interestingly, we found the MMTV-HER2/MMTV-MED1 double-transgenic mice show a significantly earlier tumor onset (~6 weeks earlier on average) when compared with that of MMTV-HER2 mice (Figure 2B). Furthermore, although most of the MMTV-HER2 mice bear only one primary tumor, most of the MMTV-HER2/MMTV-MED1 mice have two or more (Figures 2C and 2D). Moreover, tumors in MMTV-HER2/MMTV-MED1 mice exhibited greatly accelerated growth and increased weight when compared with that of controls (Figures 2E and 2F). Additionally, immunohistochemistry staining of cell proliferation marker Ki67 demonstrated that the percentage of proliferating cells in MMTV-HER2/MMTV-MED1 tumors is significantly increased when compared with that of MMTV-HER2 tumors (Figures 2G and 2H). Finally, immunocytochemistry staining (Figure S2A) and western blots (Figures S2B–S2D) confirmed the elevated MED1 and phosphorylated MED1 (p-MED1) levels in MMTV-HER2/MMTV-MED1 tumors compared with that from MMTV-HER2 controls, whereas HER2 and ERα expressions were not changed as expected. Realtime PCR analyses further indicated that HER2 mRNA expression was also not significantly affected by MED1 overexpression (Figure S2E). In addition, we examined MED1 and HER2 protein levels in these mouse tumors and several well-studied human breast cancer cells (Figures S2F–S2H). We found that the MED1 is expressed at a similarly low level in MMTV-HER2 and HER2-negative MCF7 cells compared with that of HER2-positive BT474 and SKBR3 cells. Importantly, MED1 expression in MMTV-HER2/MMTV-MED1 cells is increased to a level comparable to that of BT474 and SKBR3 cells, while the HER2 level is comparable to BT474 cells and higher than SKBr3 cells. Together, these data indicate a key role for MED1 overexpression in promoting MMTV-HER2 tumor onset and growth, and demonstrate the clinical relevance of our newly established MMTV-HER2/MMTV-MED1 mammary tumor model.

### MED1 overexpression enhances the EMT, migration, and invasion capabilities of MMTV-HER2 tumors

**Figure 3.**
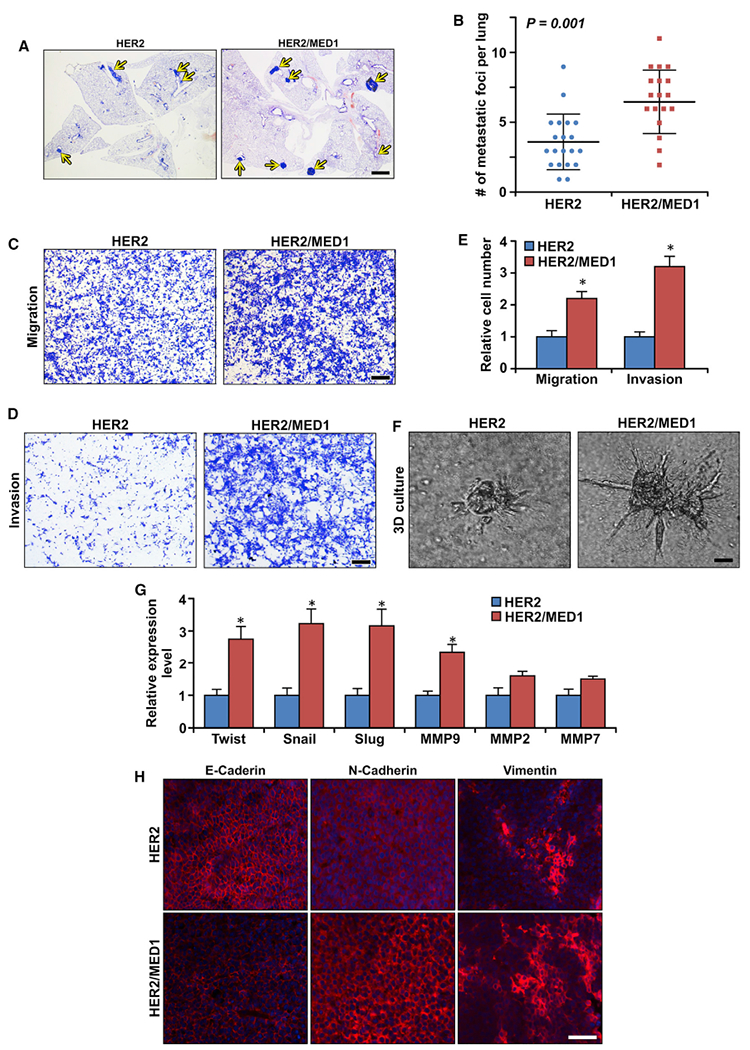
MED1 enhances MMTV-HER2 tumor cell migration, invasion, and EMT (A and B) H&E staining analyses of tumor lung metastasis in MMTV-HER2 (n = 20) and MMTV-HER2/MMTV-MED1 (n = 18) mice (A), and the quantification (B). Scale bar: 1 mm. (C and D) Transwell migration (C) and invasion (D) assays of MMTV-HER2 and MMTV-HER2/MMTV-MED1 tumor cells. Scale bar, 100 μm. (E) Quantification of (C) and (D). (F) 3D culture acini formation of MMTV-HER2 and MMTV-HER2/MMTV-MED1 tumor cells. Scale bar: 20 μm. (G) Real-time RT-PCR analyses of the expression of indicated EMT-related genes in MMTV-HER2 and MMTV-HER2/MMTV-MED1 tumors. (H) Immunofluorescence analysis of E-cadherin, N-cadherin, and Vimentin in MMTV-HER2 and MMTV-HER2/MMTV-MED1 tumor sections. Scale bar: 50 μm. The values are obtained from three independent experiments and shown as mean ± SD. *p < 0.05.

To assess whether MED1 overexpression affects tumor lung metastasis, we carried out H&E staining to analyze the metastatic lesions in the lung serial section of MMTV-HER2 and MMTV-HER2/MMTV-MED1 tumor-bearing mice. The results showed that the tumor lung metastasis was also significantly increased in the MMTV-HER2/MMTV-MED1 mice (Figures 3A and 3B). Because the increased tumor lung metastasis could be caused by the differential growth rate of the primary tumors, we next determined the migration and invasion capabilities of the tumor cells using transwell assays (Figures 3C and 3D). Our data indicated that MMTV-HER2/MMTV-MED1 tumor cells have significantly enhanced migration and invasion capabilities compared with those of MMTV-HER2 tumor cells (Figure 3E). By using a more physiological 3D culture assay, we also observed an increased escaping of MMTV-HER2/MMTV-MED1 tumor cells from the acini and invading the surrounding matrix compared with that of MMTV-HER2 cells (Figure 3F). To study whether MED1 overexpression affects tumor cell EMT (Thiery and Sleeman, 2006; Kalluri and Weinberg, 2009), we performed real-time PCR and found the expression of several EMT-related genes, including *twist*, *snail*, and *slug*, were upregulated in the MMTV-HER2/MMTV-MED1 tumors (Figure 3G). Further immunofluorescence staining confirmed the increased N-cadherin and Vimentin and decreased E-cadherin protein expression of MMTV-HER2/MMTV-MED1 tumors (Figure 3H). Because migration of tumor cells often requires the expression of matrix metalloproteinases (MMPs) to degrade the extracellular matrix (ECM) (Cui et al., 2012; Rørth, 2009), we next analyzed the expression of MMPs in these tumors. We found that, although the mRNA levels of *MMP2* and *MMP7* were not changed, *MMP9* mRNA was significantly increased in MMTV-HER2/MMTV-MED1 tumors (Figure 3G). Taken together, these data support that MED1 overexpression promotes MMTV-HER2 tumor migration, invasion, and the expression of genes involved in EMT and ECM degradation.

### MED1 overexpression expands CSCs of MMTV-HER2 tumor

**Figure 4.**
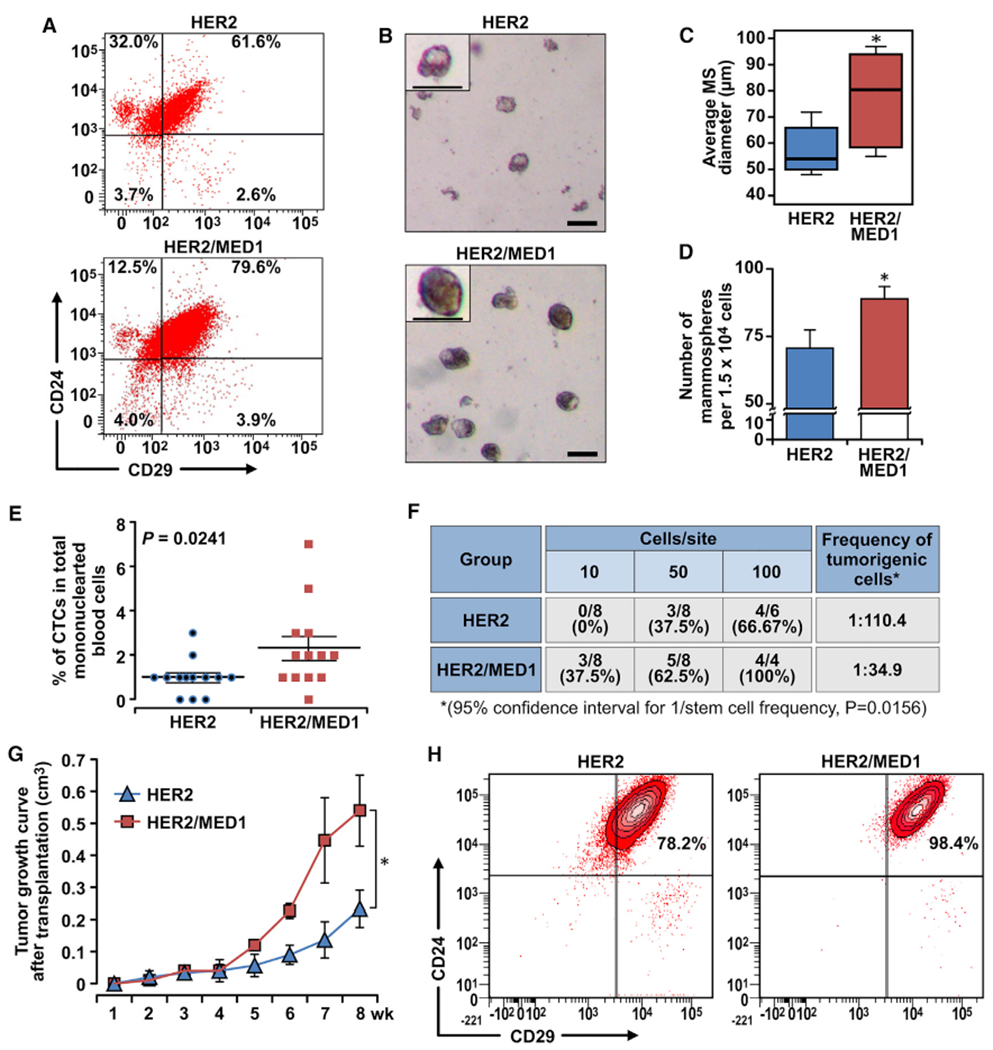
MED1 overexpression promotes MMTV-HER2 CSC formation (A) FACS analyses of MMTV-HER2 and MMTV-HER2/MMTV-MED1 CSCs using antibodies against cell surface markers Lin, CD24, and CD29. (B) Mammosphere assays using FACS-sorted tumor cells in (A). Scale bar: 100 μm. (C and D) Average diameters (C) and numbers (D) of mammospheres formed in (B). (E) Statistics of flow cytometry analysis of CD45^−^CK18^+^EpCAM^hi^ circulating tumor cells (CTCs) in mononuclear blood cells from MMTV-HER2 and MMTV-HER2/MMTV-MED1 tumor-bearing mice (n = 13). (F) Limiting dilution analyses of tumor-initiating cells in MMTV-HER2 and MMTV-HER2/MMTV-MED1 bulk tumors. (G) Growth curves of orthotopic MMTV-HER2 and MMTV-HER2/MMTV-MED1 tumor xenografts (n = 6). (H) FACS analyses of the grafted tumors using cell surface markers Lin (CD31, CD45, and Ter119), CD24, and CD29. The values are obtained from three independent experiments and shown as mean ± SD. *p < 0.05.

CSCs have been proposed as a driving force for tumorigenesis and metastatic seeding (Dalerba et al., 2007; Visvader and Lindeman, 2012). Using age-matched control, MMTV-HER2, MMTV-MED1, and MMTV-HER2/MMTV-MED1 mice, we have found a combined effect of HER2 and MED1 on mammary stem cell formation by mammosphere formation and flow cytometry assays (Figures S1H–S1K). To further determine whether HER2 and MED1 overexpression play a role in CSC formation, we first evaluated the CSCs in these tumors by fluorescence-activated cell sorting (FACS) analyses using established markers (Visvader, 2009; Shackleton et al., 2006). The data demonstrated that the Lin^−^CD24^+^CD29^+^ CSC-enriched population was significantly increased in MMTV-HER2/MMTV-MED1 tumors compared with that of MMTV-HER2 tumors (p < 0.05) (Figure 4A). We next performed mammosphere formation assays using the same number of FACS-isolated cells from each group (Figure 4B). Notably, we found that cells from MMTV-HER2/MMTV-MED1 tumors show a significant increase in both the size and the number of mammospheres formed compared with that of MMTV-HER2 tumors (Figures 4C and 4D). Moreover, flow cytometry analyses indicated an increase in the number of CTCs present in the bloodstream of MMTV-HER2/MMTV-MED1 mice (Figure 4E). Limiting dilution assays further determined there was about 1 tumor-initiating cell (TIC) in 110.4 bulk MMTV-HER2 tumor cells. However, this number was significantly increased to about 1 out of 34.9 cells in MMTV-HER2/MMTV-MED1 bulk tumors (Figure 4F). To further test the function of CSCs from these tumors *in vivo*, we transplanted FACS-sorted CSCs under the inguinal mammary fat pad of non-obese diabetic severe combined immunodeficiency (NOD-SCID) mice. As shown in Figures 4G and S2I, we found the grafted MMTV-HER2/MMTV-MED1 tumor cells grow much faster than MMTV-HER2 tumors. Consistent with these and the above results, IHC staining confirmed the increased percentage of Ki67-positive cells (Figures S2J and S2K), and FACS analyses showed a higher percentage of CSCs in the MMTV-HER2/MMTV-MED1 grafted tumors (Figure 4H). H&E staining further showed significantly increased lung metastasis in these MMTV-HER2/MMTV-MED1 CSCs grafted mice (Figures S2L and S2M). To directly examine the effect of MED1 overexpression on the colonization phase of mammary tumor metastasis, we injected tumor cells into nude mice through the tail vein. We found significantly more MMTV-HER2/MMTV-MED1 than MMTV-HER2 tumor cells formed metastatic lung nodules by H&E staining of serial lung sections (Figures S2N and S2O).

### MED1 and HER2 cooperatively regulate the migration, invasion, and stemness of mammary epithelial cells

To further evaluate whether MED1 and HER2 function together in promoting cell metastatic capabilities and stem cell formation, we isolated and transfected normal mammary epithelial cells with plasmids expressing MED1, HER2, or both MED1 and HER2. Western blot analyses confirmed the successful overexpression of these proteins in each group as expected (Figure S3A). We then carried out transwell assays (Figure S3B) and found that the number of migrated and invaded epithelial cells in the HER2-overexpression group was robustly increased when compared with that of the vector control group as expected (Johnson et al., 2010) (Figure S3C). Further, MED1 overexpression also clearly enhanced the migration and invasion of mammary epithelial cells. Significantly, overexpression of both MED1 and HER2 further promoted the migration and invasion capabilities of the mammary epithelial cells. Consistent with the results from the transwell assays, our *in vitro* mammosphere formation analysis further demonstrated that transient overexpression of MED1 or HER2 is sufficient to promote the mammosphere formation of mammary epithelial cells, while overexpression of both MED1 and HER2 can promote it to an even higher level (Figures S3D–S3F).

### MED1 overexpression affects the response of HER2-positive tumors to lapatinib treatment

**Figure 5.**
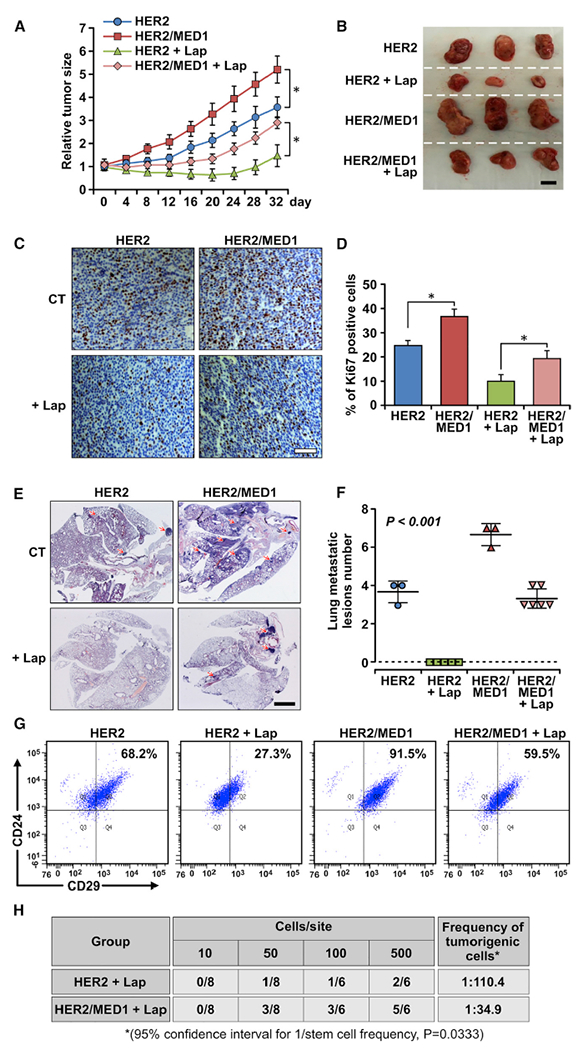
MED1 plays a key role in the response of MMTV-HER2 tumor cells to lapatinib treatment (A) Growth curve of orthotopically xenograft MMTV-HER2 and MMTV-HER2/MMTV-MED1 tumors treated with vehicle control (n = 3) or lapatinib (n = 6). (B) Representative tumor images from each treatment group at the time of collection. Scale bar: 1 cm. (C and D) IHC staining of Ki67 in tumor sections (C) and quantification (D). Scale bar: 50 μm. (E and F) H&E staining analyses of serial lung sections of above mice (E) and quantification of metastatic lesions (F). Scale bar: 2 mm. (G) Representative FACS analysis results of Lin^−^CD24^+^CD29^hi^ CSCs in vehicle- or lapatinib-treated MMTV-HER2 and MMTV-HER2/MMTV-MED1 xenograft tumors. (H) Limiting dilution analyses of bulk cells from MMTV-HER2 and MMTV-HER2/MMTV-MED1 xenograft tumors after lapatinib treatment. The values are obtained from three independent experiments and shown as mean ± SD. *p < 0.05.

A number of anti-HER2 therapeutic regimens have been developed for clinical use; however, there are currently major obstacles with these therapies that include lack of effectiveness and rapid development of resistance (Nahta et al., 2006; Chen et al., 2008; Rexer and Arteaga, 2012). To investigate whether MED1 overexpression plays a role in these processes, we examined the effect of MED1 overexpression on MMTV-HER2 tumor response to lapatinib treatment *in vivo* (Figure 5A). We found that lapatinib can inhibit orthotopically grafted MMTV-HER2 tumor growth as expected. Interestingly, however, although lapatinib can inhibit their growth, MMTV-HER2/MMTV-MED1 tumors treated with lapatinib still grow as fast as that of vehicle-treated MMTV-HER2 control tumors. Thus, the tumor size in the lapatinib-treated MMTV-HER2/MMTV-MED1 group is similar to that of the vehicle-treated MMTV-HER2 control group and is significantly larger than that of the lapatinib-treated MMTV-HER2 group at the time of tumor collection (Figure 5B). Furthermore, Ki67 staining showed there are more proliferating cells in lapatinib-treated MMTV-HER2/MMTV-MED1 tumors than that in lapatinib-treated MMTV-HER2 tumors, which is highly comparable to that of the vehicle-treated MMTV-HER2 control group (Figures 5C and 5D). Strikingly, H&E staining indicated that although lapatinib treatment can completely block MMTV-HER2 tumor cell lung metastasis, MMTV-HER2/MMTV-MED1 tumors can still actively metastasize to lung even in the presence of lapatinib, just like the vehicle-treated MMTV-HER2 control group (Figures 5E and 5F). Because CSCs have been recognized as a driving force for tumor growth, metastatic seeding, and treatment resistance (Brooks et al., 2015), we also assessed the CSC population in these tumors by flow cytometry (Figure 5G). Consistent with tumor growth and metastasis data above, we found the CSC content is significantly higher in lapatinib-treated MMTV-HER2/MMTV-MED1 tumors and is close to that of the vehicle-treated MMTV-HER2 control, which was further confirmed by mammosphere formation assays (Figures S3G–S3I). Limiting dilution analysis again showed that MMTV-HER2/MMTV-MED1 tumors still have a significantly higher number of TICs when compared with that of MMTV-HER2 tumors even after lapatinib treatment (Figure 5H). Finally, we have further extended our study to use HER2^+^ BT474 human breast cancer cells that also overexpress MED1. Consistent with the findings from our mouse model, we found that knockdown of MED1 can significantly sensitize BT474 cells to lapatinib treatment (Figure S4A) and reduce their mammosphere formation capabilities (Figures S4B–S4D). Taken together, these data support a key role for MED1 overexpression in regulating CSCs maintenance and resistance to anti-HER2 treatment.

### Gene expression profiling reveals Jab1 as the key MED1 target in MMTV-HER2 tumors

**Figure 6.**
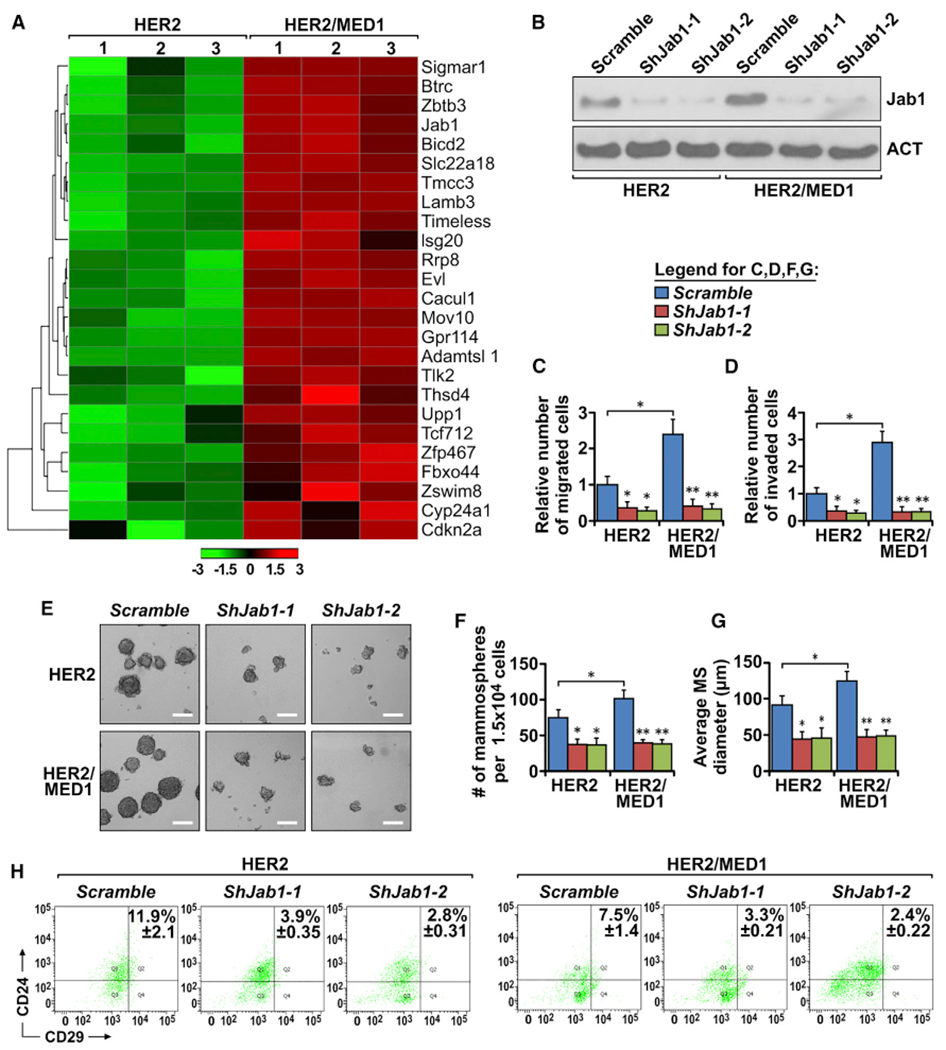
Jab1 is a direct target of MED1 overexpression involved in metastasis and CSC formation of HER2-driven tumors (A) Heatmap of top upregulated genes in MMTV-HER2/MMTV-MED1 tumors compared with that in MMTV-HER2 controls by RNA-seq (expression-based classification, p < 0.05). (B) Western blot analysis of Jab1 expression of MMTV-HER2 and MMTV-HER2/MMTV-MED1 cells treated with lentiviruses expressing control *Scramble* or two independent *shRNAs against Jab1*. (C and D) Quantification of migrated (C) and invaded (D) MMTV-HER2 and MMTV-HER2/MMTV-MED1 tumor cells as treated in (B). (E–G) Mammospheres formation assays (E) and quantification of mammospheres number (F) and size (G) of above cells as treated in (B). Scale bar: 100 μm. (H) Representative results of flow cytometry analysis of CD24^+^CD29^hi^ CSCs in the mammospheres formed in (E). The values are obtained from three independent experiments and shown as mean ± SD. *p < 0.05, **p < 0.01.

To identify which HER2/MED1 downstream target genes contributed to the observed aggressive metastasis and CSC formation phenotypes, we performed RNA-seq gene profiling analyses using MMTV-HER2 and MMTV-HER2/MMTV-MED1 tumors (Figure 6A). Of the top 14 candidate genes upregulated 2-fold and above, we have been able to confirm the upregulation of *BTB3*, *ZBTB3*, *Tmcc3*, *EVL*, *Jab1*, *Rrp8*, *Gpr114*, *Tcf7l2*, and *Adamtsl1* genes at a mRNA level by real-time RT-PCR analysis (Figure S5A). To further determine which of these are direct targets of MED1, we performed chromatin immunoprecipitation (ChIP) assays and found the recruitment of MED1 to the promoter region of *Evl* and *Jab1* genes was significantly increased in MMTV-HER2/MMTV-MED1 tumors when compared with that from MMTV-HER2 controls (Figure S5B). Western blot analysis further showed that the Jab1, but not EVL, protein level was significantly and constantly upregulated in MMTV-HER2/MMTV-MED1 tumors (Figures S5D and S5E). To further test the function of Jab1, we knocked down its expression in MMTV-HER2 and MMTV-HER2/MMTV-MED1 tumor cells by lentiviruses expressing two independent short hairpin RNAs (shRNAs) against Jab1 (Figure 6B). We found that knockdown of Jab1 by either shRNA was able to significantly reduce the cell migration and invasion of these cells (Figures S5F and S5G; Figures 6C and 6D). In addition, CSC formation of these tumor cells was also severely inhibited by Jab1 knockdown, as indicated by mammosphere assays (Figures 6E–6G) and flow cytometry analyses (Figure 6H).

Next, we overexpressed Jab1 in MMTV-HER2 cells by transient transfection of EGFP-Jab1- or control EGFP-overexpressing plasmids. Immunoprecipitation (IP) and western blots analyses showed that the total MED1 protein level was decreased, while its ubiquitination level was increased by Jab1 overexpression in these cells, as expected (Figures S6A and S6B). Further, transwell assays demonstrated the migration and invasion capabilities of EGFP-Jab1-overexpressing MMTV-HER2 cells were similar to those of MMTV-HER2/MMTV-MED1 cells and much higher than those of EGFP-overexpressing MMTV-HER2 control cells (Figures S6C and S6D). Similarly, both mammosphere formation and flow cytometry analyses indicated an increased stem cell formation of EGFP-Jab1-overexpressing MMTV-HER2 cells to a level comparable to that of MMTV-HER2/MMTV-MED1 cells (Figures S6E–S6H). Taken together, these results support that *Jab1* is a key MED1 downstream direct target gene that plays critical roles in metastasis and CSC maintenance of HER2^+^ breast tumors.

### Interplay between MED1 and Jab1 regulates ER-target gene expression in HER2-positive breast cancer

**Figure 7.**
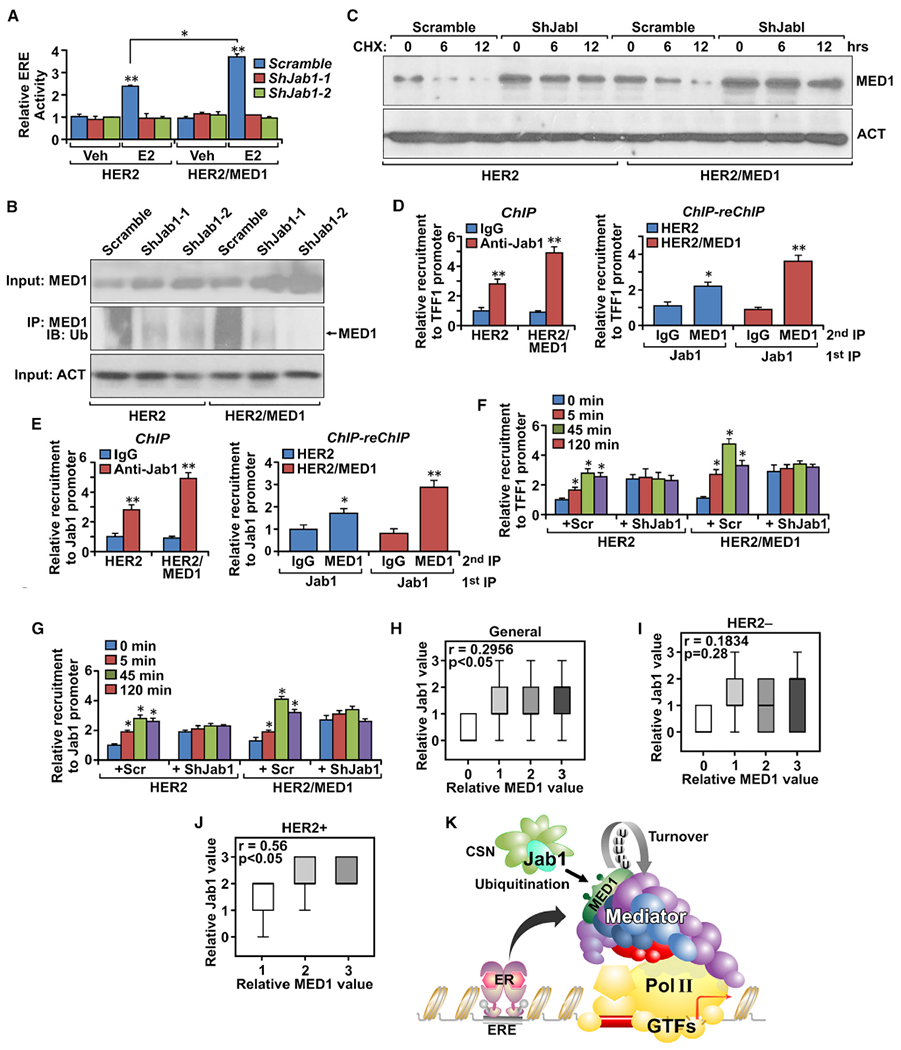
Jab1 positively regulates MED1 transcriptional function by mediating its ubiquitination status (A) ERE-luciferase assays of MMTV-HER2 and MMTV-HER2/MMTV-MED1 tumor cells transfected with *Scramble* or two independent *shRNAs against Jab1*. (B) Immunoprecipitation and western blots analysis of MED1 ubiquitination in MMTV-HER2 and MMTV-HER2/MMTV-MED1 tumor cells transfected with *Scramble* or two independent *shRNAs against Jab1*. Total MED1 and Actin proteins in the input were also analyzed. (C) Western blot analysis of MED1 protein level in MMTV-HER2 and MMTV-HER2/MMTV-MED1 tumor cells transfected with *Scramble* or two independent *shRNAs against Jab1* and treated with cycloheximide (50 μg/mL) for indicated durations. (D and E) ChIP and ChIP-reChIP analyses of Jab1 recruitment and its co-existence with MED1 at the promoter region of two MED1 target genes *TFF1* (D) and *Jab1* (E). (F and G) ChIP analyses of MED1 promoter recruitment on *TFF1* (F) and *Jab1* (G) genes using MMTV-HER2 and MMTV-HER2/MMTV-MED1 tumor cells transfected with *Scramble* or *shRNA against Jab1*. (H–J) Correlation of MED1 with Jab1 protein levels in the total (n = 52) (H), HER2^−^ (n = 37) (I), and HER2^+^ (n = 15) (J) breast cancer clinical samples. (K) Diagram for the interplay between MED1 and Jab1. The values are obtained from three independent experiments and shown as mean ± SD. *p < 0.05, **p < 0.01.

To further understand the molecular mechanism underlying Jab1 functions in MMTV-HER2/MMTV-MED1 tumors, we first examined whether Jab1 plays a role in ER-mediated gene transcription. Interestingly, we found that estrogen-stimulated estrogen response element (ERE)-luciferase reporter gene expression was completely blocked by Jab1 knockdown (Figure 7A). Consistent with that, real-time PCR analyses confirmed a requirement of Jab1 for the expression of endogenous ER target genes, such as *TFF1*, *Cyclin D1*, and *c-Myc* as well (Figure S7B). We next examined whether dual knockdown of Jab1 and MED1 will further decrease ERE-luciferase activities in human breast cancer BT474 cells (Figure S7A). We found that the knockdown of either MED1, Jab1, or both all resulted in a similar level of inhibition of ERE-luciferase activities. Because Jab1 is a key catalytic component of constitutive photomorphogenesis 9 (COP9) signalosome complex (CSN) that mainly regulates protein proteasome degradation, we then checked the ubiquitination status of MED1 and found significantly decreased MED1 ubiquitination levels after Jab1 knockdown (Figure 7B). Similarly, knockdown of two other CSN subunits, CSN2 and CSN3, also led to decreased MED1 ubiquitination and increased MED1 protein levels in these cells (Figure S7C).

Furthermore, when we examined protein steady-state levels and stabilities, we found significantly increased MED1 protein expression and enhanced stability in Jab1 knockdown cells when compared with that of control-treated cells (Figure 7C). To further examine whether MED1 and Jab1 work together on the promoter of ER target genes, we carried out ChIP-reChIP assays and confirmed their co-occupancies on the ER target genes, including *TFF1*, *Jab1*, and *Cyclin D1* (Figures 7D and 7E; Figure S7D). To understand the role of Jab1 on the dynamic promoter recruitment of MED1 at these target gene promoters, we carried out time-course experiments to examine the estrogen-induced cyclic recruitment of MED1 on ER target gene promoters (Figures 7F and 7G; Figure S7E). Interestingly, we found that although the basal level of MED1 on the ER target gene is enhanced in Jab1 knockdown cells, the estrogen-induced cyclic recruitment of MED1 is completely blocked. Finally, we performed IHC staining to analyze the expression of MED1 and Jab1 proteins in human breast cancer clinical samples and found a significantly positive correlation between MED1 and Jab1 proteins levels (Figure 7H; Figure S7F). Importantly, further analyses indicate that their significant positive correlation exists only in the HER2^+^ (Figure 7J), but not in the HER2^−^, patient population (Figure 7I). Taken together, these results support Jab1 as a direct downstream transcriptional target of MED1 in HER2^+^ breast tumors that can also reciprocally regulate MED1 ubiquitination and cyclic promoter recruitment (Figure 7K).

## DISCUSSION

Despite co-amplification and clinical correlation of MED1 with HER2 in human breast cancer, the role of MED1 in HER2-driven tumorigenesis remains largely unknown. In this study, we have generated MED1 mammary-specific overexpression transgenic mice and crossed them with the well-established MMTV-HER2 tumor mouse model. We found the following: (1) MED1 overexpression only modestly accelerates pubertal mammary gland development and does not induce tumor formation by itself; (2) MED1 overexpression significantly promotes HER2-driven breast tumor initiation and progression with increased tumor multiplicities; (3) MED1 overexpression enhances MMTV-HER2 tumor cell EMT; (4) MED1 overexpression increases HER2-positive tumor CSC/TIC formation; (5) genome-wide analysis revealed Jab1 as a key MED1 target gene, and interplay between MED1 and Jab1 contributes to HER2^+^ cancer cell migration, invasion, and CSC maintenance; and (6) Jab1 can also reciprocally regulate MED1 transcriptional activities through promoting its ubiquitination-mediated turnover and cyclic recruitment at the target gene promoters.

Overall, our study provided evidence that a gene localized in the HER2-amplicon plays critical roles in HER2-driven breast tumorigenesis *in vivo*. The *HER2/neu* gene is localized to the chromosome *17q12* region that contains at least 10 genes, including *CAB-1*, *DARPP-32*, *PNMT*, *TRAP100*, and *TRAP220/MED1* (Kauraniemi et al., 2001; Luoh, 2002; Sahlberg et al., 2013). Although some of these genes, such as *DARPP-32* (Hamel et al., 2010) and *GRB7* (Giricz et al., 2012), have already been reported to be associated with poor prognosis and survival of cancer patients, there is currently limited information available on whether any of these genes may play a role in HER2-driven breast tumor formation or treatment resistance. Our recent study has confirmed the correlation of MED1 protein levels with HER2 status using human breast cancer tissue microarrays (Cui et al., 2012). We further reported a causal relationship between HER2 overexpression and MED1 phosphorylation, promoter recruitment, and activation of ER target genes in mediating anti-estrogen resistance (Cui et al., 2012). Here, through generation of a MED1 mammary gland-specific overexpression mouse model, we further provided strong evidence that MED1 overexpression can promote HER2-driven tumor onset, growth, metastasis, and resistance to anti-HER2 treatment.

We found that MED1 overexpression greatly promotes MMTV-HER2 mammary tumor formation by as early as 6–8 weeks, although MED1 overexpression itself only modestly affects mammary gland ductal growth during the early pubertal stage and does not induce hyperplasia or tumor formation. Consistent with that, we found increased cell proliferation, EMT, and its related genes expression (e.g., twist, snail, slug, E-cadherin, and Vimentin) in MMTV-HER2/MMTV-MED1 tumors when compared with that of MMTV-HER2 tumors. In addition, we found MED1 overexpression promotes the CSC formation and desensitizes the responses of MMTV-HER2-driven tumors to anti-HER2 treatment. This is important because recent studies support a key role for CSCs in driving breast tumorigenesis, recurrence, and metastasis (Kakaralaand Wicha, 2008; Dalerba et al., 2007). MMTV-HER2 tumors are generally considered to originate from luminal progenitor cells and therefore are mostly ER^+^, albeit with gradual loss of ERα expression later, but the tumors still maintain growth dependence on and respond to estrogen stimulation (Yang et al., 2003;Shyamala et al., 2006). MED1 is mainly expressed, like ER, in luminal epithelial cells (Jiang et al., 2010), and consistent with that, we found that MED1 overexpression is able to increase the Lin^−^CD24^+^CD29^lo^ luminal stem/progenitor and ER^+^ (Lin^−^CD24^+^Scal^+^) progenitor cell populations during pubertal development. Significantly, the CSC formation in MMTV-HER2/MMTV-MED1 tumors was significantly increased as indicated by flow cytometry analyses of the Lin^−^CD24^+^CD29^hi^ CSC-enriched population, mammosphere formation assays, CTC analyses, and *in vivo* orthotopic xenograft assays. Our limiting dilution assays using bulk tumor cells further showed that the CSC/TIC population in MMTV-HER2/MMTV-MED1 tumors was significantly higher than that in MMTV-HER2 tumors.

Mechanistically, we have provided further evidence supporting Jab1 as the direct transcriptional target gene of the HER2/MED1 axis contributing to the observed aggressive phenotypes of MMTV-HER2/MMTV-MED1 tumors by RNA-seq transcriptome and subsequent analyses. Jab1, also known as CSN5, is a component of CSN previously reported to be involved in the regulation of cell proliferation, cell-cycle progression, and tumorigenesis (Shackleford and Claret, 2010; Wu et al., 2009). Importantly, knockdown of Jab1 severely impaired the migration and invasion, as well as the stem cell formation, of MMTV-HER2/MMTV-MED1 tumor cells. Furthermore, Jab1 overexpression significantly enhanced the MMTV-HER2 tumor cells in these capacities, as well as that of the MMTV-HER2/MMTV-MED1 level. Moreover, our analyses of clinical samples further found high nuclear Jab1 and MED1 signals and their positive correlation in HER2^+^ breast tumor tissues. Interestingly, we found that not only is Jab1 a direct target of MED1, but Jab1 can also function reciprocally to regulate ER-mediated transcription and MED1 functions. We found that knockdown of Jab1 greatly blocked the expression of both ERE-luciferase reporter and endogenous ER target genes. Although our study focused on the effects of Jab1 on the ER-mediated functions and we found that several non-ER target genes were not affected by Jab1 (Figure S5C), we cannot exclude the possibility that Jab1 could also contribute to HER2^+^ breast cancer through non-ER-dependent pathways.

Interestingly, we found that Jab1 works in conjunction with MED1 on the ER target gene promoter by regulating MED1 turnover and cyclic recruitment. It seems paradoxical that Jab1 knockdown stabilizes MED1, and Jab1 overexpression reduces MED1 protein through ubiquitination, but ER-mediated transcription was blocked and enhanced, respectively. However, it is known that estrogen-bound ERα recruits diverse transcriptional cofactors to its target gene promoter in a cyclic fashion to regulate their transcription, but the exact nature and functional significance of this cyclic recruitment of cofactors are just starting to be understood (Yi et al., 2017; Shang et al., 2000). Our results are consistent with the notion that cyclic recruitment of MED1 is essential for ER-mediated transcription through ubiquitin-mediated degradation to initiate a new round of cofactor recruitment and transcription. This could be similar to the previously reported essential role of ER degradation for its transcription activities, although it is not clear whether its cyclic recruitment was also affected (Bick et al., 2019). There are other ER coactivators, such as SRC-1 and CBP, also subject to degradation through the ubiquitin-proteasome pathway, but their intrinsic transcriptional activity is not affected when the proteasome is inhibited (Lonard et al., 2000). Thus, our study provides potential insights linking proteasome-dependent ER-mediated transcription to the regulation of stability and cyclic recruitment of transcriptional coactivator MED1 for proper execution of ER-mediated transcription.

Major obstacles current anti-HER2 therapies are facing are a lack of effectiveness and rapid development of resistance (Nahta et al., 2006; Rexer and Arteaga, 2012). Here, we found that MED1 overexpression plays a critical role in regulating the response of HER2^+^ tumors to lapatinib treatment. Our data indicate that, in spite of treatment, MMTV-HER2/MMTV-MED1 tumors grow as fast as vehicle-treated MMTV-HER2 controls. More strikingly, lapatinib can effectively block the MMTV-HER2 tumor lung metastasis *in vivo*, but MMTV-HER2/MMTV-MED1 tumors metastasize to lung even with lapatinib treatment and, again, as efficiently as the vehicle control-treated MMTV-HER2 group. We have recently shown that HER2/MED1 crosstalk plays a key role in the resistance of ER^+^ tumors to anti-estrogen regiments, such as tamoxifen and fulvestrant (Cui et al., 2012; Zhang et al., 2013). In this study, we further extended our previous findings by showing that MED1 overexpression can also render resistance to anti-HER2 treatments. Together, our data support MED1 as a major player in breast cancer resistance to both HER2- and ER-targeted therapies, and that MED1 and its target genes, such as Jab1, could represent promising targets to overcome the resistance of HER2^+^/ER^+^ tumors to these therapies. We have further shown that dual treatment with tamoxifen and lapatinib exhibited the greatest inhibition on HER2^+^ tumor cell growth compared with single treatments of either tamoxifen or lapatinib (Figure S3J). Moreover, emerging clinical studies have started to suggest the combined use of anti-HER2 and anti-estrogen therapies, which could provide a more favorable treatment outcome for this subtype of tumor (Prat and Baselga, 2013; Montemurro et al., 2013). Thus, simultaneous targeting of MED1and ER/HER2 signaling pathways could enhance therapeutic response and improve outcome for the particularly challenging ER/HER2 double-positive luminal B breast cancers. Importantly, our MMTV-HER2/MMTV-MED1 mouse model reported here will not only allow for better understanding of the molecular mechanisms of HER2/MED1 in tumorigenesis but also offer a more clinically relevant tumor model where both HER2 and MED1 are overexpressed for preclinical trials.

## STAR★METHODS

### RESOURCE AVAILABILITY

#### Lead contact

Further information and request for resources and reagents should be directed to and will be fulfilled by the Lead Contact, Dr. Xiaoting Zhang.

#### Materials availability

All unique/stable reagents generated in this study are available from the Lead Contact with a completed Materials Transfer Agreement.

#### Data and code availability

The RNA-Seq data generated during this study are available at GEO repository (GEO: GSE148922: https://www.ncbi.nlm.nih.gov/geo/query/acc.cgi?acc=GSE148922).

### EXPERIMENTAL MODEL AND SUBJECT DETAILS

#### Mice

MMTV-MED1 mice were generated by pronuclear microinjection of pMMTV-MED1 plasmids at the University of Cincinnati Transgenic Mouse Core Facility. Transgenic progeny was identified by Southern blot and PCR analyses. MMTV-HER2 mice (Guy et al., 1992) were purchased from The Jackson Laboratory. To monitor primary mammary tumor formation, manual palpation was performed weekly and the tumor volume was calculated by the formula: volume = length × width^2^ /2. Tumor-free survival was analyzed using the Kaplan-Meier method. All animals were housed in AAALAC-approved facilities at the University of Cincinnati. All procedures were approved by IACUC and in accordance with the NIH guidelines outlined in the Guide for Care and Use of Laboratory Animals. All mice used in this breast cancer study are female mice with an age range from 6 weeks up to 18 months. For orthotopic tumor xenograft experiments, freshly isolated bulk mammary tumor cells (limited dilution assay) or 1 × 10^5^ flow cytometry sorted CSCs-enriched cells were suspended with PBS and mixed with matrigel (1:1) to a final volume of 100 μl. The mixture was injected into the fourth mammary fat pads of 6-week-old NOD-SCID or Nude mice (Jackson Laboratories) as described (Pece et al., 2010). Tumor sizes were measured with calipers weekly as above and the frequencies of TICs in limiting dilution assays were calculated using the following website: http://bioinf.wehi.edu.au/software/elda/. For tail vein injection, 5 × 10^5^ MMTV-HER2 and MMTV-HER2/MMTV-MED1 tumor cells were suspended in 100 μl PBS and injected through 6-week-old nude mice tail vein using sterile 27-gauge noodle. The mice were maintained for another 3 weeks before euthanization and lung collection. For lapatinib treatment, when the tumor volume reached 600 mm^3^, mice were randomly assigned to vehicle (0.5% hydroxypropylmethylcellulose (Sigma) with 0.1% Tween 80 (Sigma) in water) or lapatinib (100 mg/kg body weight, LC Laboratories) treatments by oral gavage five days per week for 5 weeks. At the end of treatment, tumors were collected for flow cytometry analysis, mammosphere culture assays as well as paraffin/frozen sections for immunostaining analysis. Lung tissues from the tumor bearing mice were also collected and processed for H&E staining and metastasis analysis.

#### Cell lines and primary culture

Mammary epithelial cells were isolated as previously described (Smalley et al., 2012). In brief, the fourth pubertal mammary glands were collected and then cut into fine pieces using a razor blade following lymph node removal. The resulting tissue was suspended in DMEM/F12 medium containing 2 mg/ml collagenase and incubated at 37°C for 45 min with gentle agitation. The tissue was pelleted by centrifugation at 400 g for 5 min, re-suspended in 0.25% trypsin and incubated at 37°C for 10 min. The organoids were washed, filtered through 40 μm cell meshes, suspended in DMEM/F12 (1:1) medium containing 10% fetal bovine serum and cultured in a 6-well-plate for one hour. The floating cells were then transferred to a new cultured dish and cultured for further FACS analyses and lentivirus infection. MMTV-HER2 and MMTV-HER2/MMTV-MED1 tumor cells were prepared as previously described (Yang et al., 2018) and cultured in DMEM (high glucose) supplemented with 10% FBS. BT474, MCF-7, and SKBr3 cells were obtained from ATCC and maintained in DMEM supplemented with 10% FBS.

#### Human breast cancer tissue samples

The human breast cancer specimens were obtained from the University of Cincinnati Cancer Institute (UCCI) Breast Cancer Center of Excellence. These samples were collected from female breast cancer patients with an age range of 24-89 years and processed in compliance with protocol #09-04-14-02 approved by the Institutional Review Board (IRB) of the University of Cincinnati.

### METHOD DETAILS

#### Antibodies, plasmids and lentivirus packaging

The following antibodies were used for immunostaining experiments at indicated dilution: rabbit anti-MED1 (1:1500) (Cui et al., 2012); rabbit anti-Ki67 (1:250, Thermo Scientific, clone SP6); rabbit anti-HER2 (1:200, CST); rabbit anti-p27 (1:1000, CST); rabbit anti-E-Cadherin (1:200, Abcam); rabbit anti-N-Cadherin (1:200, Abcam); rabbit anti-Vimentin (1:200, Abcam) and rat anti-mouse CD31 (1:150, BD PharMingen™). The following antibodies were used for western blots analyses at indicated dilution: rabbit anti-Actin (1:10,000, Sigma); rabbit anti-MED1(1:1500; Zhang et al., 2005b); rabbit anti-p-MED (1:1000) (Cui et al., 2012); rabbit anti-HER2 (1:1000, CST); rabbit anti-p-HER2 (Tyr1248) (1:1000, CST); rabbit anti-ERα (1:500, Santa Cruz, clone HC-20); mouse anti-Jabl (1:500, Santz Cruz sc-13157); mouse anti-Timeless (1:500, Santz Cruz sc-393122) and mouse anti-Evl (1:500, Santa Cruz, sc-365751). The following antibodies were used for flow cytometry analysis at indicated dilution: rat anti-mouse CD45-APC (1:100, BioLegend); rat anti-mouse TER-119-APC (1:100, BD PharMingen ™); rat anti-mouse CD31-APC (1:100, BD PharMingen ™); rat anti-mouse CD24-PE (1:100 BD PharMingen ™); hamster anti-mouse CD29-FITC (1:100, BD Biosciences); mouse anti-Cytokeratin Pan-Fluor®488 (1:100, Thermo Fisher) and anti-mouse CD326 (EpCAM)-PE (1:100, Affymetrix eBioscience). Doxycycline (Fisher Scientific) was dissolved in distilled water, filter sterilized, aliquoted and stored at −20°C in the dark and used at indicated concentration.

The pCDH-CMV-MED1 construct has been generated previously (Cui et al., 2012). To generate a lentiviral construct expressing HER2, the target segment was amplified by PCR using pcDNA3-HER2 plasmid (Addgene plasmid #16257) as the template. The PCR product was digested with restriction enzymes *Nhe* I and *Not* I, cloned into lentiviral vector pCDH-CMV (System Biosciences) and the sequence was confirmed by sequencing (Genewiz). Jab1 overexpressing plasmid (Plasmid #111213) was purchased from Addgen. Two clones (TRC #304598 and 331796) of verified mouse specific pLKO.1-shJabl, pLKO.1-shCSN2 (TRC #125057), pLKO.1-shCSN3 (TRC #349924) and scrambles bacterium stocks were purchased from Sigma. High titer lentiviruses were generated by transient co-transfection of 293T cells vector only, pLKO.1-Scramble, pLKO.1-shJab1, pLKO.1-shCSN2, pLKO.1-shCSN3, pCDH-CMV-MED1 or pCDH-CMV-HER2 with packaging constructs pMD2.G and psPAX2 according to the manufacturer’s instructions (System Biosciences).

#### Mammosphere, transwell and 3-D culture assays

For the mammosphere formation assay, 3 × 10^4^ cells were seeded into each well of ultra-low adhesion 24-well plates (Coring) in DMEM/F-12 (1:1) medium (Corning) containing 0.4% BSA (Fisher BioReagents), Insulin (5 ng/ml, Sigma), B-27 (50x, Invitrogen), EGF (20 ng/ml, R&D) and bFGF (10 ng/ml, R&D) and 1% Penicillin/Streptomycin (Fisher BioReagents). Mammospheres were imaged 7-10 days after initiation of culture and analyzed using Zeiss Axiovision software (Carl Zeiss, Jena, Germany). For invasion and migration assays, 5 × 10^4^ serum-starved tumor cells were suspended with 100 μl serum-free medium and plated in 8 μm pore Transwell inserts (Corning, NY) in a 24-well plate format with or with 100 μl matrigel (Corning, 1:10 diluted in DMEM), respectively. The lower compartment was filled with 700 μl medium containing 10% FBS and cultured for 18 hr. Cells were then fixed and stained in 20% methanol containing 1% crystal violet for 10 min. Tumor cell 3-D culture was conducted essentially as described in Lee et al. (2007). In brief, 100 μl of matrigel was spread evenly with a pipette tip on the surface of pre-chilled 24-well plate and incubate at 37^°^C for 30 min to allow the matrigel to gel. 5× 10^3^ cells were suspended in 600 μl mammosphere culture medium with 5% matrigel, added onto the pre-coated surface and cultured for 14 days with the medium changed every 4 days.

#### Tissue processing and immunostaining

Whole mount mammary glands were stained as described (Jiang et al., 2010). Briefly, the forth mammary gland were isolated, spread on glass slides and fixed in Carnoy’s fixative (6 parts 100% ethanol, 3 parts CHCl3 and 1 part glacial acetic acid) overnight. The fixed tissues were rehydrated in a series of 70%, 50% and 30% ethanol for 15 min each and finally rinsed in distilled water for 5 min. The tissues were stained with Carmine Alum Stain (1g carmine (Sigma) and 2.5g aluminum potassium sulfate (Sigma) per 500 mL distilled water) overnight, dehydrated in a series of 50%, 70% and 100% ethanol, cleared in xylene and mounted with Permount (Vector Laboratories). Tumor and lung tissues were fixed in 10% natural buffered formalin (Fisher Scientific) overnight and transferred to 70% ethanol the next day. Samples were then paraffin embedded and cut at 5 mm per section. For lung metastasis analysis, serial sections of all five lobes were stained by H&E and counted for all the metastatic lesions. For immunostaining, following heat-induced antigen retrieval (10 min in a pressure cooker) in citrate buffer (10mM Citric Acid, 0.05% Tween 20, pH 6.0), tissue sections were treated with 1% H_2_O_2_ in methanol for 30 min to block the endogenous peroxidase activity and then blocked with 5% goat serum in TBST followed by overnight incubation with primary antibodies at 4°C. Sections were incubated for 1 hr with the appropriate biotin-conjugated (1:2000, Jackson ImmunoResearch) or fluorescence conjugated secondary antibodies. For IHC staining, the VECTASTAIN Elite ABC kit (Vector Laboratories) were used following the instructions and the slide was developed using a DAB kit (Vector Laboratories). Images were taken and processed with an axioplan imaging 2e microscope (Zeiss). For immunofluorescence staining, Nuclear DNA were stained with DAPI. Wimasis image analysis software was used to calculate the microvessel density.

#### FACS analysis

Mammary epithelial and tumor cells were subjected to FACS analysis as described (Shackleton et al., 2006; Luo et al., 2013). Briefly, cell suspensions were incubated on ice for 30 min with different combinations of the following antibodies: PE-CD24; FITC-CD29; FITC-Sca-1; APC-CD31; APC-CD45 and APC-Ter119. To analyze the CTCs, the mononuclear cells were prepared as described in Lu et al. (2010). In brief, 300μl of blood from tumor bearing mice were collected through submandibular vein and the red blood cells were lzyed in lysis buffer (155 mM NH4Cl, 12 mM NaHCO3 and 0.1 mM EDTA), the remaining mononuclear cells were fixed in Acetone at −20°C for 10 min and cells were then labeled with 488-CK18, APC-CD45 and PE-EpCAM antibodies. Cells were sorted using a FACSCanto II system (BD Biosciences) and the data were analyzed with FACSDiva 6.1.1 software.

#### Cycloheximide treatment, IP and western blot

Cycloheximide was dissolved in culture medium at a final concentration of 50μg/ml and the cells were cultured for indicated duration. The cells were then collected and protein prepared for western blots analysis. For immunoprecipitation (IP), cells were lysed in RIPA buffer and incubated for overnight at 4°C with anti-MED1 antibody followed by 1 hr incubation with Protein A/G Agarose beads. The precipitated protein was then went through western blots analysis using anti-ubiquitin primary antibody. For western blots, tumor tissues or cells were lysed in RIPA buffer (50mM Tris pH 8.0, 150mM NaCl, 1% Triton X-100, 0.5% Sodium deoxycholate, 0.1% SDS, 2mM EDTA, 5% Glycerol, protease inhibitors cocktail (Roche), 0.1M DTT and 0.1M PMSF) for 30 min on ice and centrifuged for 10 min at 12,000 g. Supernatants were collected, and protein concentration was measured using a standard Bradford assay. 30μg of each extract were subjected to SDS-PAGE using 10% gels and transferred to nitrocellulose membranes. The membranes were blocked with 5% fat free milk in TBST for 1 hr at room temperature and incubated with primary antibodies at 4°C overnight. After incubating with HRP-conjugated secondary antibodies (1:5000, Jackson ImmunoResearch) for 2 hr, the signals were detected by PicoWest Chemiluminescent reagent (Pierce).

#### RNA-seq and Heatmap generation

RNA sequencing libraries were generated by using a TruSeq stranded mRNA library prep kit and sequenced using Illumina HisEq 2000. Single-end RNA-seq reads were mapped to the reference mouse genome (mm10) using TopHat v.2.1.0 (Trapnell et al., 2012). Transcript quantification and differential expression analysis were performed by Cufflinks v2.2.1 and Cuffdiff 2, respectively (Trapnell et al., 2012). For differentially expression analysis, the fold-change cutoff was set at 1.5 or higher. Benjamini-Hochberg false discovery rate adjusted P value less than 0.05 was considered statistically significant. Heatmaps were generated by GENE-E (https://software.broadinstitute.org/GENE-E/index.html) using RPKM output from Cufflinks. Rows were clustered using Pearson correlation metric and average linkage.

#### Chromatin Immunoprecipitation

ChIP assays were performed as previously described (Cui et al., 2012; Zhang et al., 2013). In brief, cells were fixed with formaldehyde at a final concentration of 1% for 10 min at 37°C. After washing with ice-cold PBS, cells were harvested, lysed and sonicated to generate an average DNA size of 500 bp. Immunoprecipitation experiment were then carried out using indicated antibodies and the immunoprecipitated chromatin then went through reverse cross-linking at 65°C for 6 hr or reChIP. In reChIP experiments, the precipitated chromatins were eluted by incubation at 37°C for 30 min in 10mM DTT. The supernatant was then diluted 20 times with ChIP buffer and subject to the ChIP procedure again using the indicated antibodies. The final recovered DNA was analyzed through real-time PCR using the primers were listed in Table S1.

#### RNA preparation and real-time RT-PCR

Total RNA was extracted using RNeasy Mini Kit (QIAGENe) and RNA concentrations were measured using the NanoDrop ND-1000. For real-time RT-PCR, 2 μg of total RNA were used for reverse transcription using a SuperScript III first strand synthesis system (In-vitrogen). Real time PCR was performed using 2 x SYBR Green PCR Master Mix reagents (Roche) on an ABI Prism 7700 Sequence Detection System (Applied Biosystems). Primers for the PCR reactions were shown in Table S1 (Jiang et al., 2010).

#### ERE-luciferase Assays

ERE-luciferase assay was carried as described (Cui et al., 2012). Briefly, MMTV-HER2, MMTV-HER2/MMTV-MED1 and BT474 cells were infected with scramble control, sh-Jab1 or ShMED1 lentivirus that specifically knockdown Jab1 or MED1. Cells were then cultured in 24-well plates containing phenol-red-free DMEM medium supplemented with 5% charcoal-stripped FBS for 48 hours. ERE-TK-Luc reporter vector and pRL-CMV control plasmid were transfected using Lipofectamine 2000 (Invitrogen). Following the transfection, the cells were treated with estrogen or vehicle for 24 hours before harvest. A dual luciferase reporter assay system (Promega) was used to measure the luciferase activity.

### QUANTIFICATION AND STATISTICAL ANALYSIS

All experiments were repeated at least 3-5 times and data was expressed as average ± SD. Statistical analyses of the data were performed by pairwise Student’s t test. It is considered statistically significant (*) if p ≤ 0.05 and very significant (**) if P ≤ 0.01. Kaplan-Meier tumor free survival data were compared using the log-rank test. Tumor number and metastatic lesions were statistically analyzed using GraphPad software with two-tailed Student’s t tests.

## Supplementary Material

1

2

## Figures and Tables

**Table:** KEY RESOURCES TABLE

REAGENT or RESOURCE	SOURCE	IDENTIFIER
Antibodies		
Rabbit polyclonal anti-MED1	(Zhang et al., 2005a)	N/A
Rabbit monoclonal anti-Ki67	Thermo Scientific	Cat # MA5-14520; RRID: AB_10979488
Rabbit monoclonal anti-HER2	Cell Signaling	Cat# 2165; RRID: AB_560966
Rabbit monoclonal anti-p27	Cell Signaling	Cat# 3686; RRID: AB_2077850
Rabbit polyclonal anti-E-Cadherin	Abcam	Cat# ab15148; RRID: AB_301693
Rabbit polyclonal anti-N-Cadherin	Abcam	Cat# ab18203;RRID: AB_444317
Rabbit monoclonal anti-Vimentin	Abcam	Cat# ab92547; RRID: AB_10562134
Rat monoclonal anti-Mouse CD31	BD Biosciences	Cat# 550274; RRID: AB_393571
Mouse monoclonal anti-β-Actin	Sigma-Aldrich	Cat# A2228; RRID: AB_476697
Phospho-MED1	Cui et al., 2012	N/A
Phospho-HER2 (Tyr1221/1222)	Cell Signaling	Cat # 2243; RRID: AB_490899
Rabbit polyclonal anti-ERα	Santa Cruz	Cat# sc-542; RRID: AB_631470
Mouse monoclonal anti-Jab1	Santz Cruz	Cat# sc-13157; RRID: AB_627835
Mouse monoclonal anti-Timeless	Santz Cruz	Cat# sc-393122; RRID: AB_2889040
Mouse monoclonal anti-Evl	Santz Cruz	Cat# sc-373794; RRID: AB_10917737
Rat anti-mouse CD45-APC	BioLegend	Cat # 103111; RRID: AB_312976
Rat anti-mouse TER-119-APC	BD PharMingen ™	Cat# 557909; RRID: AB_398635
Rat anti-mouse CD31-APC	BD PharMingen ™	Cat# 551262; RRID: AB_398497
Rat anti-mouse CD24-PE	BD PharMingen ™	Cat# 553262; RRID: AB_394741
Hamster anti-mouse CD29-FITC	BioLegend	Cat #102205; RRID: AB_312882
Mouse anti-Cytokeratin Pan-Fluor®488	Thermo Fisher	Cat # 53-9003-82; RRID: AB_1834350
Anti-mouse CD326 (EpCAM)-PE	BioLegend	Cat # 118205; RRID: AB_1134176
Anti-Mouse IgG-HRP	Cell Signaling	Cat # 7076; RRID: AB_330924
Anti-Rabbit IgG-HRP	Cell Signaling	Cat # 7074; RRID: AB_2099233
Goat anti-Rabbit FITC	Jackson ImmunoResearch Labs	Cat #111-095-144; RRID: AB_2337978
Biological samples		
Human breast cancer samples	University of Cincinnati Cancer Institute (UCCI)	University of Cincinnati Medical Center | UC Health | A Premiere Teaching Hospital
Chemicals, peptides, and recombinant proteins		
4-hydroxy-tamoxifen (4-OHT)	Sigma	Cat # 579002
Lapatinib	Selleckchem	Cat # S2111
Cycloheximide (CHX)	Sigma	Cat # C7698
RIPA buffer	Sigma	Cat # C2978
Anti-protease mix	Thermo Scientific	Cat # PI78415
SYBR Green	Bio-rad	Cat # 1725121
Collagenase	Thermo Scientific	Cat #17100017
BCA assay	Thermo Scientific	Cat # 23225
RNAeasy kit	QIAGEN	Cat # 74004
DAB Substrate Kit	Vector Laboratories	SK-4100
VECTASTAIN® ABC Kits	Vector Laboratories	PK-6100
SuperSignal West Pico PLUS Chemiluminescent Substrate	Thermo Scientific	Cat # 34579
Deposited data		
Raw and analyzed RNA-Seq data	This paper	GEO: GSE148922: https://www.ncbi.nlm.nih.gov/geo/query/acc.cgi?acc=GSE148922
Experimental models: cell lines		
MMTV-HER2	This paper	N/A
MMTV-MED1	This paper	N/A
MMTV-HER2/MMTV-MED1	This paper	N/A
MCF-7	ATCC	ATCC® CRL-3435
BT-474	ATCC	ATCC® HTB-20
SKBr3	ATCC	ATCC® HTB-30
Experimental models: organisms/strains		
MMTV-Neu	The Jackson Laboratory	Stock #: 002376
MMTV-MED1	This paper	N/A
Oligonucleotides		
Primers for Realtime PCR and ChIP analysis, see Table S1	This paper	N/A
Recombinant DNA		
pCDH-CMV-MED1	Cui et al., 2012	N/A
pCDH-CMV-HER2	This paper	N/A
pLKO.1-shJabl	Sigma	TRCN0000304598
pLKO.1-shJabl	Sigma	TRCN0000331796
pLKO.1-Scramble	Sigma	SHC016
pLKO.1-shJabl	Sigma	TRCN0000343837
pLKO.1-shCOPS2	Sigma	TRCN0000125057
pLKO.1-shCOPS3	Sigma	TRCN0000349924
pLKO.1-shMed1	Cui et al., 2012	N/A
pMD2.G	Cui et al., 2012	Addgene Cat # 12259
psPAX2	Cui et al., 2012	Addgene Cat # 12260
EYFP-JAB1	This paper	Addgene Cat #111213
pERE-tk-Luc	Cui et al., 2012	N/A
pRL-CMV	Cui et al., 2012	N/A
Software and algorithms		
ImageJ	Commercial	https://imagej.nih.gov/ij
FlowJo	Commercial	https://www.flowjo.com/
FACSDiva 6.1.1	Commercial	BD FACSDiva Software | BD Biosciences-US
TICs calculation	Online	http://bioinf.wehi.edu.au/software/elda/

## Highlights

- Generation of a more clinically relevant mouse HER2^+^/MED1^+^ mammary tumor model
- MED1 overexpression promotes HER2^+^ tumor onset, stem cell formation, and metastasis
- Critical roles of MED1 overexpression in HER2^+^ tumor treatment resistance
- Jab1 is a MED1 direct target gene mediating the aggressive phenotypes of HER2^+^ tumors
