# Supplementary material for: Functional cooperation between co-amplified genes promotes aggressive phenotypes of HER2-positive breast cancer
Source: Cell Rep. Author manuscript; Available in PMC 2021 Apr 16. (PMC8050805; doi:10.1016/j.celrep.2021.108822)
Supplement: 1 [file NIHMS1683100-supplement-1.pdf]

Cell Reports, Volume *34*

Supplemental information

Functional cooperation between co-amplified genes promotes aggressive phenotypes of HER2-positive breast cancer

Yongguang Yang, Marissa Leonard, Zhenhua Luo, Syn Yeo, Gregory Bick, Mingang Hao, Chunmiao Cai, Mahmoud Charif, Jiang Wang, Jun-Lin Guan, Elyse E. Lower, and Xiaoting Zhang

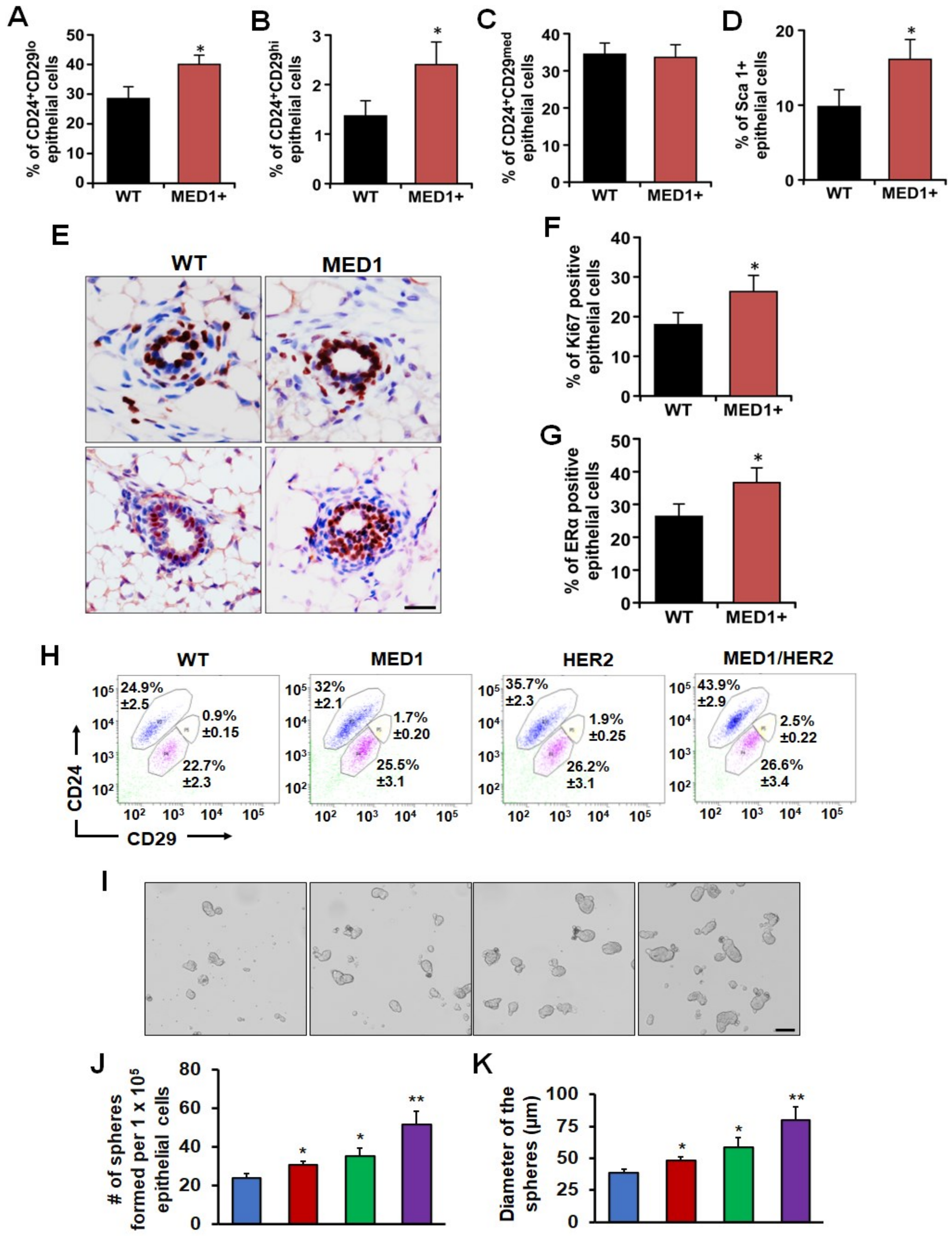

Figure S1. MED1 overexpression plays a role in mammary stem/progenitor cell formation in vivo. Related to Figure 1. (A-D) Quantification of flow cytometry analysis of $Lin^{-}CD24^{+}CD29^{lo}$ (A), $Lin^{-}CD24^{+}CD29^{hi}$ (B), Lin^{-} $CD24^{+}CD29^{mid}$ (C) and Lin^{-} $CD24^{+}Sca\ 1^{+}$ (D) cells in the mammary glands of 7-week-old control and MMTV-MED1 transgenic mice. (E) Immunocytochemistry staining of Ki67 (upper panel) and ERα (lower panel) of 7-week-old control or MMTV-MED1 transgenic mouse mammary glands. Bar = 20 μm. (F-G) Quantification of Ki67 (F) and ERα (G) positive epithelial cells in (E). (H) Flow cytometry analysis of Lin^{-} $CD24^{+}CD29^{lo}$, Lin-$CD24^{+}CD29^{hi}$ and Lin^{-} $CD24^{+}CD29^{mid}$ mammary epithelial cells from 18 -week-old wildtype control, MMTV-MED1, MMTV-HER2 and MMTV-HER2/MMTV-MED1 transgenic mice. (I-K) Mammosphere formation assays using mammary epithelial cells from 18-week-old wildtype control, MMTV-MED1, MMTV-HER2 and MMTV- HER2/MMTV-MED1 transgenic mice (I) and statistics of the number (J) and size (K) of the mammospheres formed in (I). Bar = 100 μm. The values are obtained from three independent experiments and shown as mean ± SD. *P < 0.05 or **P < 0.01.

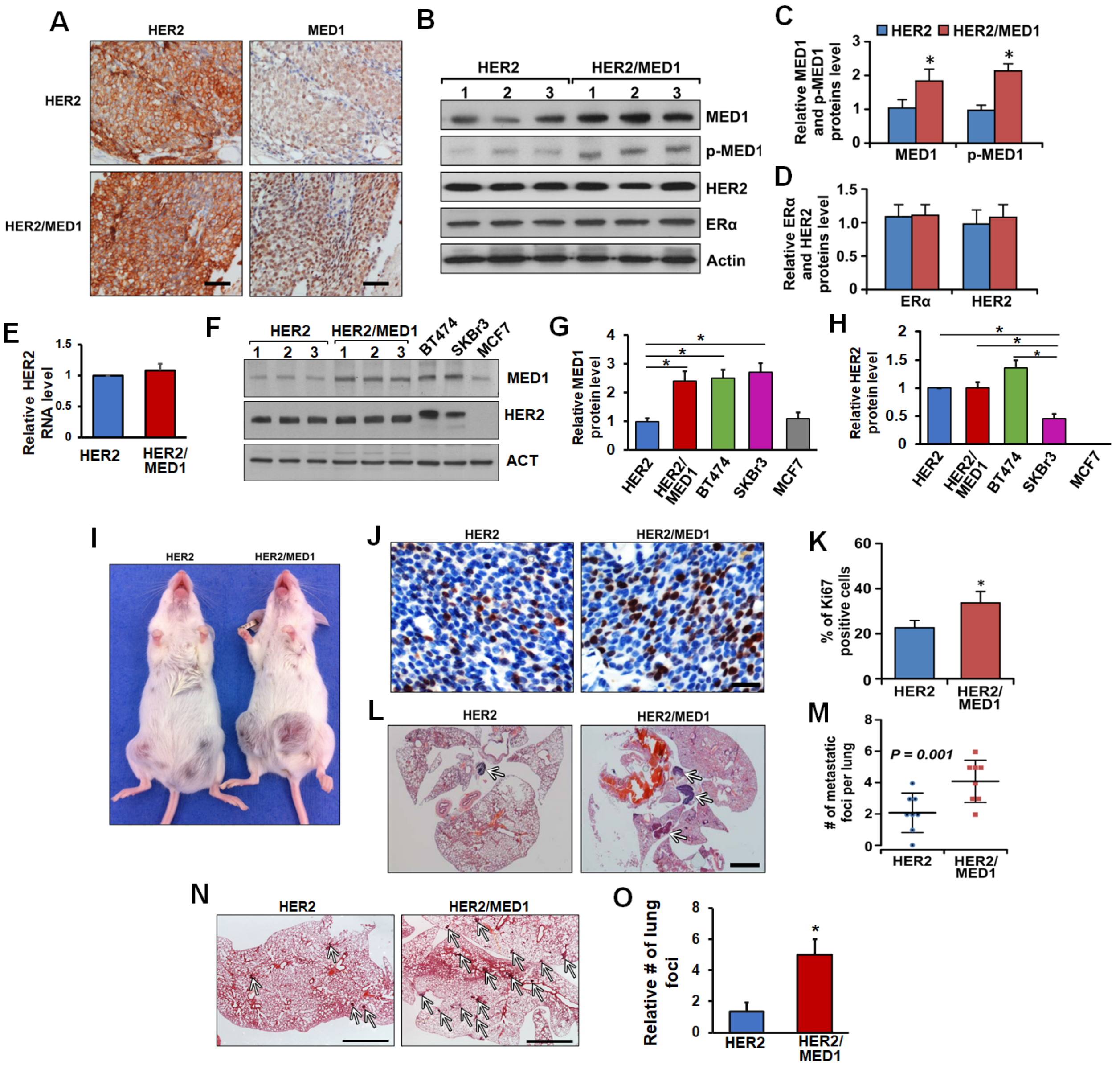

Figure S2. MED1 overexpression promotes MMTV-HER2 xenograft tumor growth and lung metastasis. Related to Figure 2. (A) IHC analyses of HER2 and MED1 expression in MMTV-HER2 and MMTV-HER2/MMTV-MED1 tumor sections. Bar = 100 µm. (B) Immunoblotting analyses of MED1, p-MED1, HER2 and ERα protein levels in MMTV-HER2 and MMTV-HER2/MED1 tumors. (C-D) Qualification of relative ERα and HER2 protein levels (C) and MED1 and p-MED1 protein levels (D) in (B). (E) Realtime PCR analysis of HER2 expression in MMTV-HER2 and MMTV-HER2/MMTV-MED1 tumor. (F-H) Western blots analysis of MED1 and HER2 expression in mouse MMTV-HER2 and MMTV-HER2/MMTV-MED1 tumors and human BT474, SKBr3 and MCF7 breast cancer cell lines (F) and quantification of relative protein level of MED1 (G) and HER2 (H). (I) Representative pictures of NOD-SCID mice bearing MMTV-HER2 or MMTV-HER2/MMTV-MED1 tumors xenografts (n=6). (J-K) IHC staining analyses of Ki67 positive cells in MMTV-HER2 and MMTV-HER2/MMTV-MED1 xenograft tumor sections (J) and quantification (K). Bar = 50 µm. (L-M) H&E staining of recipient mice lung sections (L) and quantification of metastatic lesions (M). (N-O) H&E staining of lung sections of the nude mice tailed vein injected with MMTV-HER2 and MMTV-HER2/MMTV-MED1 tumor cells (N) and quantification of relative lung foci in each group (n=5). Bar = 100 µm. (O). The values are obtained from three independent experiments and shown as mean ± SD. *P < 0.05 or **P < 0.01.

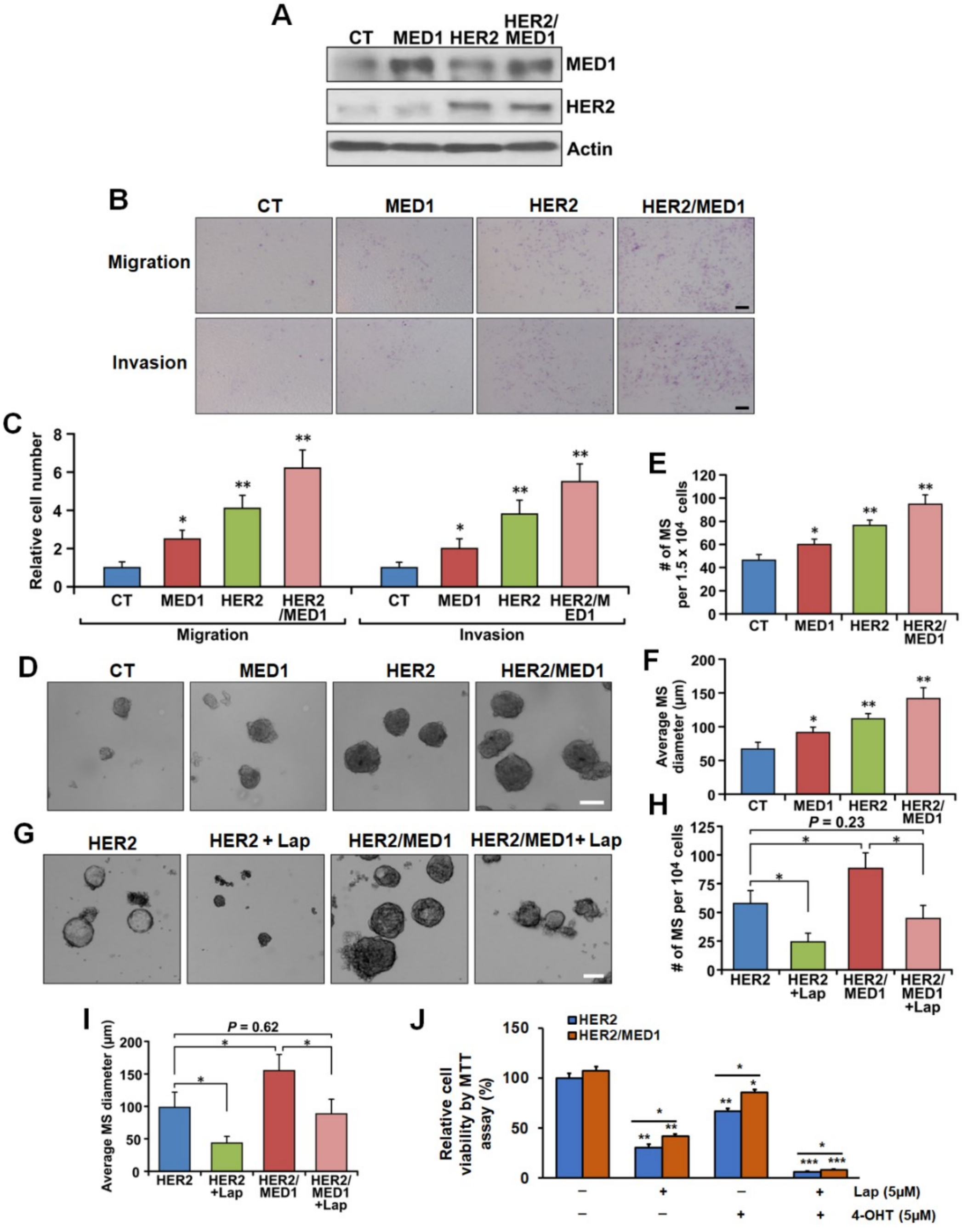

Figure S3. MED1 and HER2 cooperatively regulate mammary epithelial cell migration, invasion and stem cell formation. Related to Figure 3. (A) Western blots analyses of MED1 and HER2 expression in mammary epithelial cells infected with lentivirus expressing control, MED1, HER2, or MED1/HER2. (B-C) Transwell assays of mammary epithelial cells overexpressing control, MED1, HER2, or HER2/MED1 (B) and qualification of migrated and invaded cells (C). Bar = 100 μm. (D) Mammosphere formation of mammary epithelial cells overexpressing control, MED1, HER2, or HER2/MED1. (E-F) Qualification of the average number (E) and size (F) of mammospheres formed in (D). (G) Representative pictures of mammospheres formed by Lin-CD24 +CD29hi CSCs from vehicle or lapatinib treated MMTV-HER2 and MMTV-HER2/MMTV-MED1xenograft tumors. (H-I) Quantification of mammospheres number (H) and size (I) in (G). (J) MTT assays analysis of relative viability of MMTV-HER2 and MMTV-HER2/MMTV-MED1 cells treated with lapatinib, 4-OHT or lapatinib plus 4-OHT. Bar = 100 μm. The values are obtained from three independent experiments and shown as mean ± SD. *$P < 0.05$ or **$P < 0.01$.

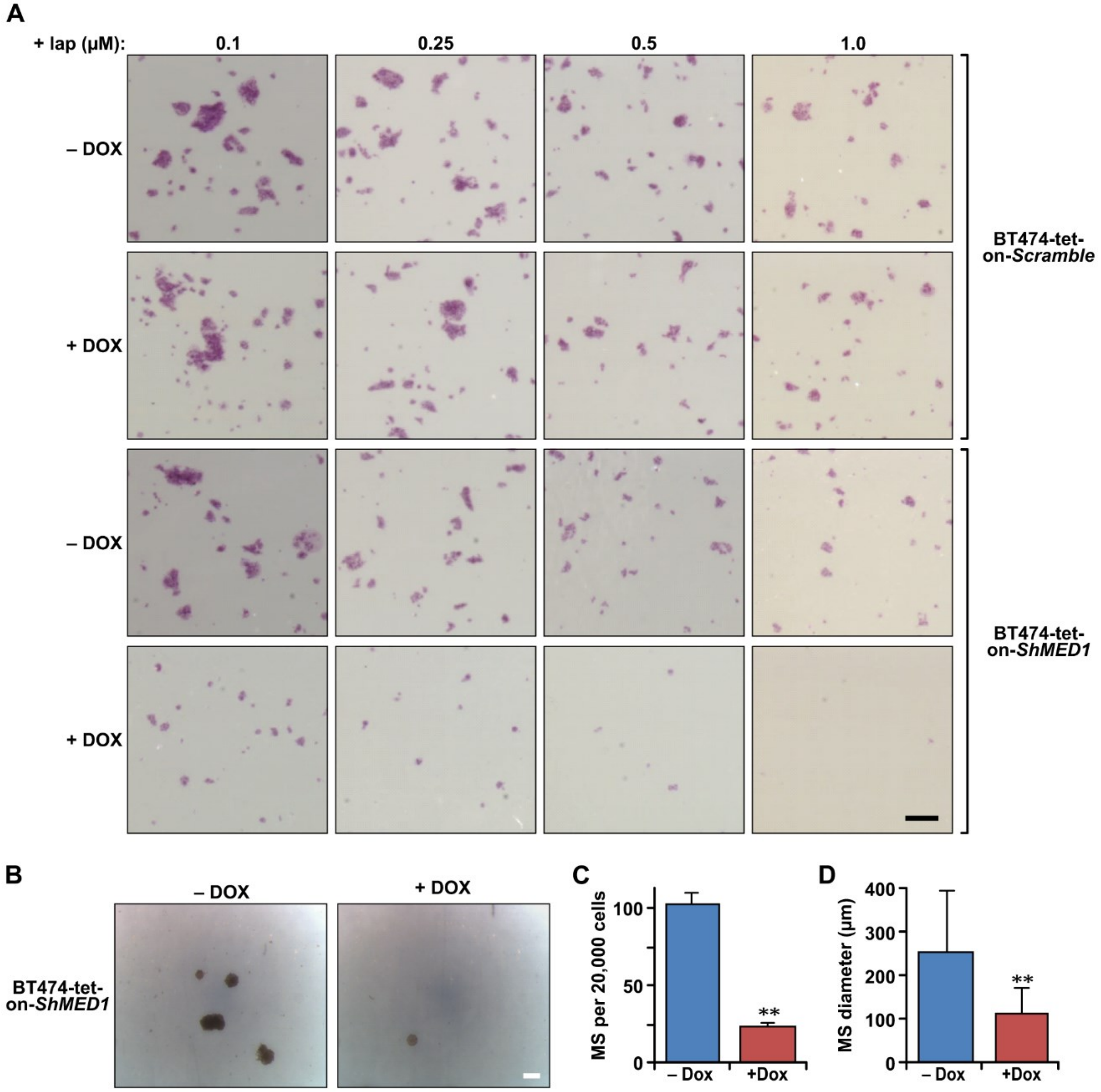

Figure S4. MED1 plays a role in mammosphere formation and lapatinib sensitivity of HER2+ human breast cancer cells. Related to Figure 5. (A) Crystal violet staining of BT474-tet-on-shMED1 and BT474-tet-onscramble cells treated with lapatinib, in the presence or absence of doxycycline (DOX). (B) Mammosphere formation assays of BT474-tet-on-shMED1 and BT474-tet-on-scramble cells in the presence or absence of doxycycline (DOX). (C-D) Quantification of mammospheres number (C) and size (D) in (B). Bar = 100 μm. The values are obtained from three independent experiments and shown as mean ± SD. *P < 0.05 or **P < 0.01.

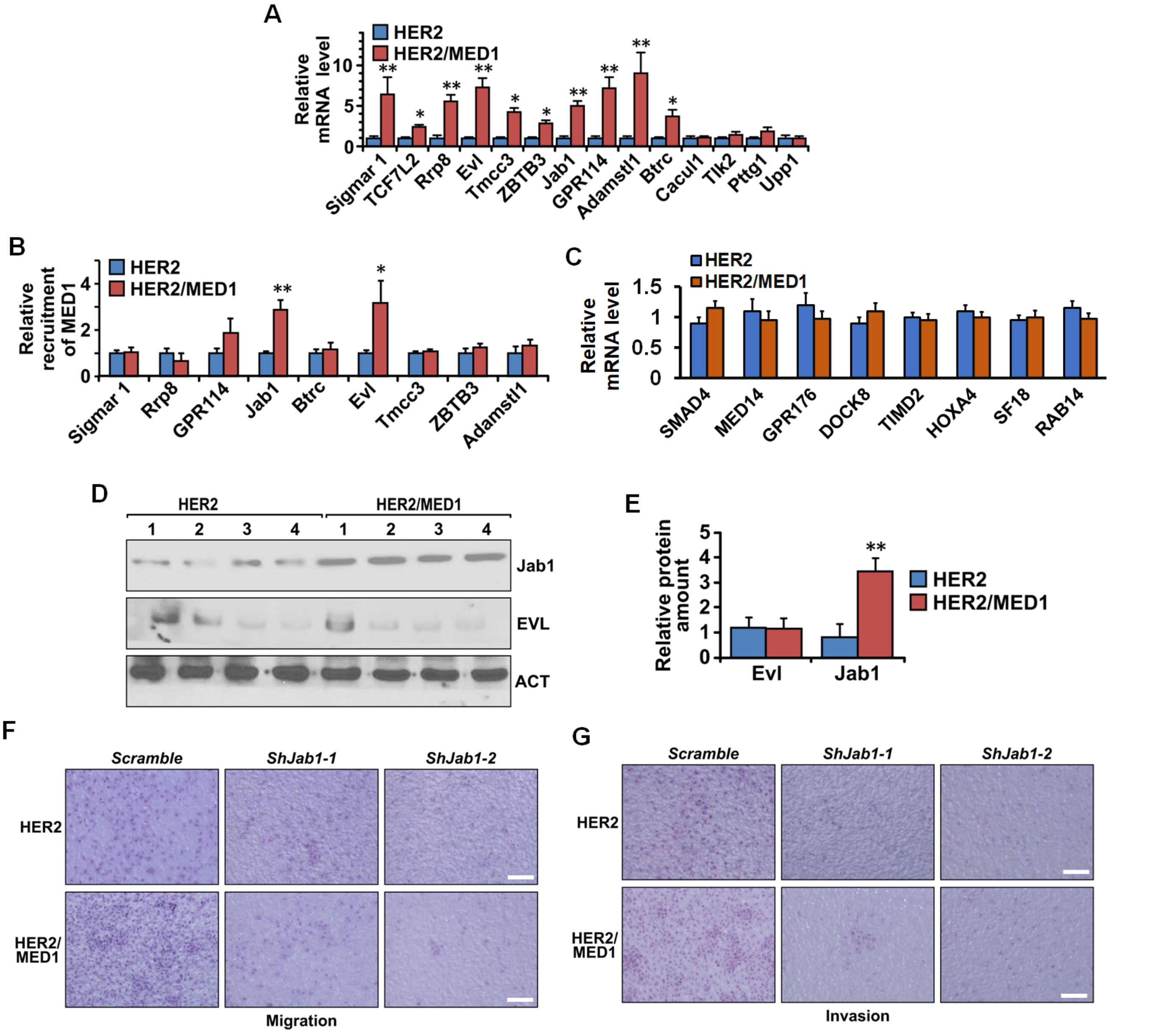

Figure S5. Jab1 is a direct target of MED1 in MMTV-HER2 tumor cells. Related to Figure 6. (A) Realtime RT-PCR analyses of top candidate genes identified by RNA-seq using MMTV-HER2 and MMTV-HER2/MMTV-MED1 tumors. (B) ChIP analyses of MED1 promoter recruitment on the confirmed upregulated genes from (A) using MMTV-HER2/MMTV-MED1 tumors cells. (C) Realtime PCR verification of indicated genes expression in MMTV-HER2 and MMTV-HER2/MMTV-MED1 tumor cells that are non-differentially expressed by RNA-Seq analysis. (D-E) Western blots analysis of Jabl and Evl expression in MMTV-HER2 and MMTV-HER2/MMTV-MED1 tumors (D) and quantification (E). (F-G) Migration (F) and invasion (G) assays of MMTV-HER2 and MMTV-HER2/MMTV-MED1 tumor cells infected with lentivirus expressing control Scramble or shRNAs against Jab1. Bar = 100 µm. The values are obtained from three independent experiments and shown as mean ± SD. *P < 0.05 or **P < 0.01.

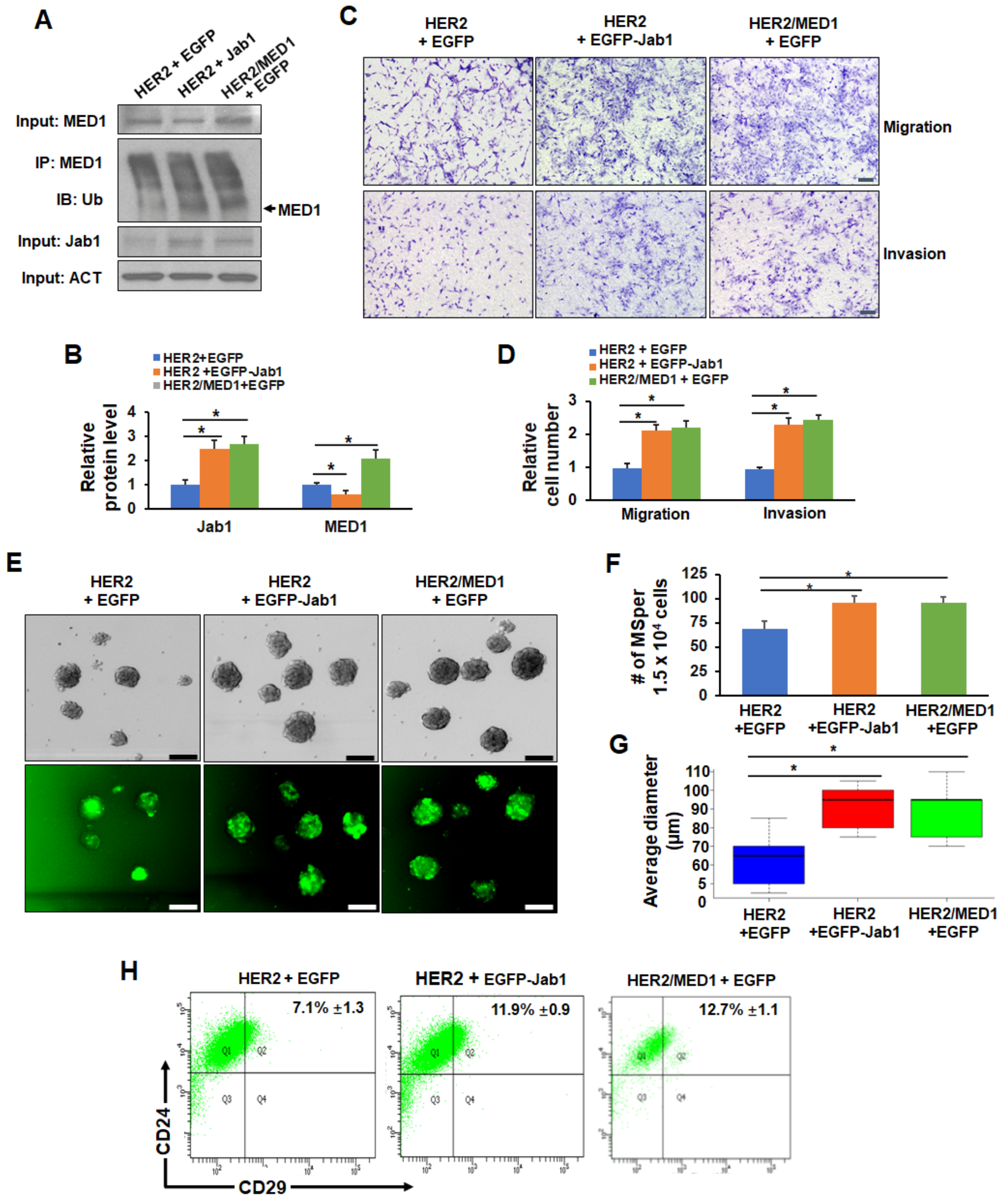

Figure S6. Jab1-overexpression enhances the migration, invasion and CSCs formation capacities of MMTV-HER2 tumor cells. Related to Figure 6. (A-B) Immunoprecipitation and western blots analysis of MED1 ubiquitination in EGFP-Jab1 or EGFP overexpressing MMTV-HER2 and MMTV-HER2/MMTV-MED1 tumor cells (A) and qualification of relative Jab1 and MED1 protein level (B). Total MED1 and Actin proteins in the input were also analyzed. (C-D) Transwell assay analysis of the migration and invasion capacities of EGFP-Jab1 or EGFP overexpressing MMTV-HER2 and MMTV-HER2/MMTV-MED1 tumor cells (C) and quantification of the relative number of migrated and invaded cells in each group (D). Bar = 100 μm. (E-G) Mammosphere formation assays using EGFP-Jab1 or EGFP overexpressing MMTV-HER2 and MMTV-HER2/MMTV-MED1 tumor cells (E), statistics of average numbers (F) and diameters (G) of mammospheres formed in (E). (H) Flow cytometry analysis of $CD24^{+}CD29^{hi}$ CSCs enriched population in the mammosphere of (E). Bar = 100 μm. The values are obtained from three independent experiments and shown as mean ± SD. *P < 0.05 or **P < 0.01.

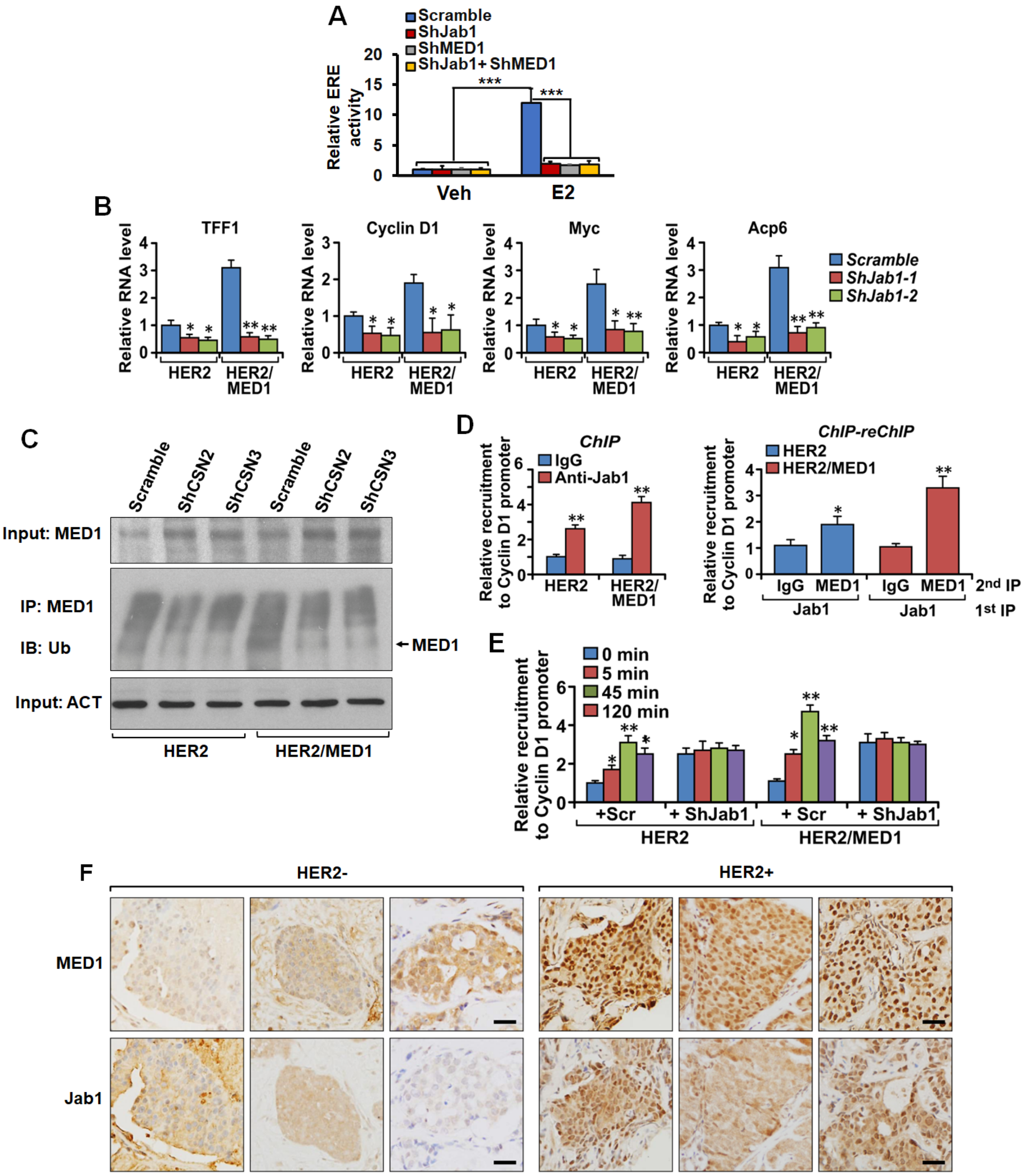

Figure S7. Jab1 positively regulates the transcriptional activity of MED1. Related to Figure 7. (A) ERE-luciferase activity assays in BT474 cells with Jab1 and MED1 single or dual knockdown treated with E2 or Vehicle (Veh) control. (B) Realtime PCR analyses of the expression of TFF1, Cyclin D1, c-Myc and ACP6 in MMTV-HER2 and MMTV-HER2/MMTV-MED1 tumor cells transfected with Scramble or two independent shRNAs against Jab1. (C) Immunoprecipitation and western blots analysis of MED1 ubiquitination in MMTV-HER2 and MMTV-HER2/MMTV-MED1 tumor cells with CSN2 and CSN3 knockdown. Total MED1 and Actin proteins in the input were also analyzed. (D) ChIP and ChIP-reChIP analyses of Jab1 recruitment and its co-existence with MED1 at the promoter region of Cyclin D1 gene. (E) ChIP analyses of MED1 promoter recruitment on Cyclin D1 gene using MMTV-HER2 and MMTV-HER2/MMTV-MED1 tumor cells transfected with Scramble or shRNA against Jab1. (F) Representative immunocytochemical analyses of the expression of MED1 and Jab1 in the sections of HER2- (left panel) (n = 37) and HER2+ (right panel) (n = 15) clinical breast cancers. Bar = 50 µm. The values are obtained from three independent experiments and shown as mean ± SD. *P < 0.05 or **P < 0.01.
